# The shifted convolution problem in function fields

**DOI:** 10.1007/s00208-026-03340-9

**Published:** 2026-03-19

**Authors:** Alexandra Florea, Matilde Lalín, Amita Malik, Anurag Sahay

**Affiliations:** 1 Department of Mathematics, University of California Irvine, 340 Rowland Hall, Irvine, CA 92697 USA; 2 Département de mathématiques et de statistique, Université de Montréal, CP 6128, succ. Centre-ville, Montreal, QC H3C 3J7 Canada; 3 Department of Mathematics, The Pennsylvania State University, McAllister Building, University Park, PA 16802 USA; 4 Department of Mathematics, Purdue University, 150 N. University Street, West Lafayette, IN 47907 USA

## Abstract

We study the shifted convolution problem for the divisor function in function fields in the large degree limit, that is, the average value of $d(f) d(f+h)$ where *f* runs over monic polynomials in $\mathbb {F}_q[T]$ of a given degree, and *h* is a given monic polynomial. We prove an asymptotic formula in the range $\deg (h) < (2-\epsilon )\deg (f)$. We also consider mixed correlations and self-correlations of $r_\chi = 1 \star \chi$, the convolution of 1 with a Dirichlet character mod $\ell$, where $\ell$ is a monic irreducible polynomial, proving asymptotic formulae in various ranges. This includes the case of quadratic characters, which yields results about correlations of norm-counting functions of quadratic extensions of $\mathbb {F}_q[T]$. A novel feature of our work is a Voronoi summation formula (equivalently, a functional equation for the Estermann function) in $\mathbb {F}_q[T]$ which was not previously available.

## Introduction

The shifted convolution of divisor functions

1 $$\begin{aligned} \frac{1}{X}\sum _{n\leqslant X} d(n) d(n+h), \end{aligned}$$

with $h \in \mathbb {N}$ is a classical problem in the analytic theory of the integers, initially considered by Ingham [33] who proved an asymptotic formula for (1) with log-savings. Soon thereafter, Estermann [26] established a power saving asymptotic for (1) by discovering and exploiting a connection to Kloosterman sums. Bounds on Kloosterman sums have since played a crucial role in this and related problems, as we shall see below.

There has been sustained interest in an asymptotic formula for questions like (1) with uniformity in the shift *h* due to applications to moment problems, a connection due to Atkinson [4]. In this case, (1) is connected to understanding the fourth moment of the zeta function,

$$\begin{aligned} \int _T^{2T} |\zeta (\tfrac{1}{2}+it)|^4 dt. \end{aligned}$$

Indeed, Heath-Brown’s breakthrough work [32] establishing a power-saving asymptotic for the fourth moment uses as an input the asymptotic (obtained via an application of Weil’s bound for Kloosterman sums)

$$\begin{aligned} \text {(1)} = P_2(\log X;h) + O(X^{-1/6+\epsilon }) \text { uniformly for } h \leqslant X^{5/6}, \end{aligned}$$

where $P_2(t;h)$ is a polynomial of degree 2 whose coefficients depend on *h*. The leading coefficient of $P_2(t;h)$ is $\sigma _{-1}(h)/\zeta (2)$, where $\sigma _{-1}(h)=\sum _{d\mid h} d^{-1}$; the other coefficients are also explicit, though more complicated to express.

Later, Deshouillers–Iwaniec [21, 22] introduced powerful methods from the spectral theory of automorphic forms to utilize cancellation between the Kloosterman sum to different moduli, thereby improving on the point-wise use of Weil’s bound.

There are many subsequent works around this circle of ideas; the reader may consult [23, 39, 47, 48] for a more comprehensive account of the extensive literature. We content ourselves with highlighting the work of Meurman [36] who proved an asymptotic formula for (1) in the widest known range $h \ll X^{2 - \epsilon }$ (see also the work of Balkanova–Frolenkov [6], showing that the method of Motohashi [39] can yield the same result).

In this article, we investigate the analogue of (1) for the function field $\mathbb {F}_q[T]$, i.e.,

2 $$\begin{aligned} \frac{1}{q^n} \sum _{f \in \mathcal {M}_n} d(f) d(f+h), \end{aligned}$$

where here and throughout *q* is a fixed prime power, $h \in \mathcal {M}$ is a monic polynomial in $\mathbb {F}_q[T]$, *d*(*f*) counts the monic divisors of *f*, and the sum $f \in \mathcal {M}_n$ runs over the set of monic polynomials of degree *n*.

The literature on (2) is much sparser than (1) but there are still some antecedents to our work. One is interested in evaluating (2) when $q^n \rightarrow \infty$. Unlike in $\mathbb {Z}$, there are two natural regimes one can consider. For example, we may fix *n* and let $q \rightarrow \infty$. Andrade–Bary-Soroker–Rudnick [1] considered this problem (among others) and demonstrated that, provided $\deg (h) < n$,

3 $$\begin{aligned} \bigg (\frac{1}{q^n} \sum _{f \in \mathcal {M}_n} d(f)d(f+h)\bigg )\Bigg / \bigg (\frac{1}{q^n} \sum _{f \in \mathcal {M}_n} d(f) \bigg )^2 \longrightarrow 1 \text { as } q \longrightarrow \infty . \end{aligned}$$

See also Gorodetsky–Sawin [29] who consider more general convolutions and observe a lower order term in the large *q*-limit yielding more information about the arithmetic factor. For a fixed *q* (or indeed, for $\mathbb {Z}$), the analogous quantity to (3) would instead depend on the prime factorization of *h*, demonstrating some local dependence between the multiplicative structures of *f* and $f+h$. Thus, (3) may be interpreted as saying that the additive arithmetic structure disappears in the large field limit.

The complementary regime of *q* fixed and $n \rightarrow \infty$ is more reminiscent of the integer setting and is the subject of our investigations. In this case, several independent authors (Conrey–Florea in unpublished work, Gorodetsky in his PhD thesis [28, Lemma 3.3], Woo in her undergraduate thesis [50], and Yiasemides in [51]) established an exact formula for (2) provided that $\deg (h) < n$. Ultimately, this relies on the fact that (the function field analogue of) the Estermann function is a polynomial. In particular, its functional equation is not used.

Our goal is to extend the range of $\deg (h)$ in which an asymptotic formula for (2) is possible (eschewing, as will be necessary, the exact formula). For a nonzero polynomial $h\in \mathbb {F}_q[T]$ denote by ${{\,\textrm{sgn}\,}}(h)$ its leading coefficient. Our main result is then the following.

### Theorem 1.1

Suppose that *h* is a polynomial with $\deg (h)=m$ and let $d =\max \{m,n\}$. Assume moreover that if $m=n$, then ${{\,\textrm{sgn}\,}}(h)\not =-1$. Then

$$\begin{aligned}&\sum _{f \in \mathcal {M}_n} d(f) d(f+h) = q^n \sum _{\begin{array}{c} g|h \\ \deg (g) \leqslant [n/2] \end{array}} \frac{1}{|g|} \Big [ 4 \deg (g)^2 \Big (1-\frac{1}{q} \Big )\\&-2\deg (g) \Big (2+\frac{2}{q}+ \Big ( 1-\frac{1}{q}\Big )(n+d)\Big )+\Big [ 2n+2 +(d-1) \Big ( n \Big ( 1-\frac{1}{q}\Big )+1+\frac{1}{q}\Big ) \Big ]\Big ]\\&\qquad \qquad \qquad -1\!\!1_{2\mid d} q^n \sum _{\begin{array}{c} g_2\mid h\\ \deg (g_2)=[n/2] \end{array}} \frac{n-2\deg (g_2)-1}{q|g_2|} + E, \end{aligned}$$

where $E=0$ if $m \leqslant n+1$ or $m=n+2$ and *n* is odd; otherwise

$$\begin{aligned} E \ll q^{m/2+\epsilon m}. \end{aligned}$$

In this result and throughout the article, implicit constants will usually depend on *q* (the size of the constant field) or $\epsilon$ but will be uniform in all other parameters. We omit the constants depending on *q* from the error terms, as we are interested in the limit where *q* is fixed and $m,n\rightarrow \infty$.

Note that this gives an asymptotic formula provided that $\deg (h) < (2-\epsilon )n$; thus, we recover exactly the same range as the aforementioned work of Meurman [36].

### Remark 1.2

Notice that when $m \leqslant n$, we have $d=n$, and we can rewrite Theorem 1.1 in the following form:

$$\begin{aligned} \sum _{f \in \mathcal {M}_n} d(f) d(f+h)&= q^n \sum _{\begin{array}{c} g|h \end{array}} \frac{1}{|g|} \Big [\Big (n-2\deg (g)+1\Big )^2-\frac{1}{q}\Big (n-2\deg (g)-1\Big )^2\Big ] +R, \end{aligned}$$

where

$$ R = {\left\{ \begin{array}{ll} 0 &  \text{ if } m < [n/2],\\ O(q^{n/2+\varepsilon n}) &  \text{ if } [n/2] \leqslant m \leqslant n. \end{array}\right. }$$

One can check that the above result agrees with the results of [28] and [50].

### Remark 1.3

The coefficient of $n^2q^n$ above is $$\left( 1-\frac{1}{q}\right) \sum _{\begin{array}{c} g|h \end{array}} \frac{1}{|g|}=\frac{1}{\zeta _{\mathbb {F}_q[T]}(2)}\sigma _{-1}(h)$$, where $\zeta _{\mathbb {F}_q[T]}(s)$ is given by (11). This corresponds to the leading coefficient of $P_2(\log X;h)$ in the number field case. Note that there is a transition in the asymptotic formula when $\deg (h) = [n/2]$ (as we have an exact formula when $\deg (h) < [n/2]$ and an error term when $[n/2] \leqslant \deg (h) \leqslant n$). Note also that there is an additional transition regime when $\deg (h)>n$, as $d =\deg (h)$ in this case in the asymptotic formula in Theorem 1.1.

We now turn to generalizations. In $\mathbb {Z}$, progress on problems like (1) goes hand in hand with similar progress about the sums-of-two-squares function

$$\begin{aligned} r(n) = \#\{(a,b) \in \mathbb {Z}^2: a^2+b^2 = n \} = 4 \sum _{d \mid n} \chi _{-4}(d). \end{aligned}$$

where $\chi _{-4}$ is the non-trivial character modulo 4. The shifted convolution of *r*(*n*) with $r(n+h)$ was first studied by Estermann [25]. Spectral methods were used by Iwaniec [34, Theorem 12.5], Chamizo [14, 15], and Aryan [3] with Chamizo achieving the best general result in the range $h\ll X^{2/(1+2\vartheta )-\epsilon }$ where $\vartheta \leqslant \frac{7}{64}$ is the best known exponent towards the Ramanujan–Petersson conjecture for Maass forms.

An analogue of sums-of-two-squares for $\mathbb {F}_q[T]$ was introduced in [10] (see also [7, 8]). For this, defining

$$\begin{aligned} r(f) = \#\{ (a,b) \in \mathbb {F}_q[T]: a^2 + Tb^2 = f\}/\{\pm 1\}, \end{aligned}$$

then *r* can similarly be written as a convolution of 1 with the quadratic character mod *T* given by the Legendre symbol $\chi (f) = \Big ( \frac{f}{T} \Big )$, see (8). Let

$$\begin{aligned} L(s,\chi ):=\sum _{f\in \mathcal {M}} \frac{\chi (f)}{q^{s\deg f}} \end{aligned}$$

be the Dirichlet *L*-function associated to $\chi$.

### Corollary 1.4

Suppose *q* is odd, *h* is a polynomial with $\deg (h)=m$ and let $d =\max \{m,n\}$. Assume moreover that if $m=n$, then ${{\,\textrm{sgn}\,}}(h)\not =-1$. Let $\chi$ be the quadratic character (i.e., the Legendre symbol) modulo *T*. Then

$$\begin{aligned} \sum _{f \in \mathcal {M}_n} r(f) r(f+h) =&\frac{ q^{n} L(1,\chi )^2}{1+q^{-1}} \sum _{\begin{array}{c} g|h \\ \deg (g) \leqslant [d/2], T \not \mid g \end{array}} \frac{ 1}{|g|} \\&- \psi _{\chi ,\chi }^\star (h) \frac{q^{n-1} L(1, \chi )^2}{1+q^{-1}} \sum _{\begin{array}{c} g| h \\ \deg (g) \leqslant [(d-1)/2], T \not \mid h/g \end{array}} \frac{ 1}{|g|}+E, \end{aligned}$$

where

$$E \ll {\left\{ \begin{array}{ll} q^{3n/4+\epsilon n} &  \text{ if } m \leqslant n, \\ q^{3\,m/4+2+\epsilon m} &  \text{ if } m>n, \end{array}\right. } $$

and

$$\psi _{\chi ,\chi }^\star (h)={\left\{ \begin{array}{ll} \chi ({{\,\textrm{sgn}\,}}(h)) &  \text{ if } m>n, \\ \chi ({{\,\textrm{sgn}\,}}(h)+1) &  \text{ if } m=n,\\ 1 &  \text{ if } m<n. \end{array}\right. }$$

Finally, note that the character in the previous result is quadratic. However, our methods work equally well for general characters. We thus prove also the following theorems, from which the above corollary immediately follows. Let

4 $$\begin{aligned} r_{\chi }(f):= \sum _{\begin{array}{c} g\in \mathcal {M}\\ g \mid f \end{array}} \chi (g) \end{aligned}$$

and let the canonical Gauss sum associated to $\chi$ be

$$G(\chi ):= \sum _{a \hspace{0.05em}(\textrm{mod}\,\ell )} \chi (a)e\Big (\frac{a}{\ell }\Big ),$$

as defined by (16). We have the following.

### Theorem 1.5

Suppose *h* is a monic polynomial with $\deg (h)=m$ and let $d =\max \{m,n\}$. Assume moreover that if $m=n$, then ${{\,\textrm{sgn}\,}}(h)\not =-1$. Let $\chi _1,\chi _2$ be two primitive characters modulo $\ell$, for some monic irreducible polynomial $\ell$ with $\deg (\ell )>0$. Then,

$$\begin{aligned}  &   \sum _{f \in \mathcal {M}_n} r_{\chi _1}(f) r_{\chi _2}(f+h) = \frac{ q^n L(1,\chi _1)L(1,\chi _2)}{L(2,\chi _1\chi _2)} \sum _{\begin{array}{c} g|h \\ \deg (g) \leqslant [d/2] \end{array}} \frac{ \chi _1 \chi _2(g)}{|g|}\\  &   + \psi _{\chi _1,\chi _2}^{*}(h) \frac{q^n \overline{\chi _2}(-1) G(\chi _1) G(\chi _2)G(\overline{\chi _1 \chi _2})L(1,\overline{\chi }_1)L(1,\overline{\chi _2})}{|\ell ^2|L(2,\overline{\chi _1 \chi _2})} \sum _{\begin{array}{c} g| h \\ \deg (g) \leqslant [(d-1)/2] \end{array}} \frac{ \chi _1 \chi _2(h/g)}{|g|}+E \end{aligned}$$

where

$$E \ll {\left\{ \begin{array}{ll} q^{3n/4+\epsilon n} &  \text{ if } m \leqslant n \text { and } \deg (\ell ) = 1, \\ q^{3d/4+2\deg (\ell )+\epsilon d} &  \text{ otherwise, } \end{array}\right. } $$

and

$$\psi _{\chi _1,\chi _2}^{*}(h)={\left\{ \begin{array}{ll} \chi _1({{\,\textrm{sgn}\,}}(h)) &  \text{ if } m>n, \\ \chi _1\chi _2({{\,\textrm{sgn}\,}}(h))\overline{\chi _2}({{\,\textrm{sgn}\,}}(h)+1) &  \text{ if } m=n,\\ 1 &  \text{ if } m<n. \end{array}\right. }$$

We note that the above is truly an asymptotic formula when $m<\frac{4n}{3}(1-\epsilon ) - \frac{8 \deg (\ell )}{3}$. It is worth remarking that the restriction that $\ell$ is prime can be removed, but doing so would be significantly more technical (see the discussion in Sect. 3.3).

### Remark 1.6

In the integer setting and with general $\chi _1,\chi _2$, work of Müller [41] obtains an asymptotic formula essentially with the shift $h\ll X^{8/9-\epsilon }$ (where the conductor of the characters is bounded by $X^{1/9-\epsilon }$). The first main term in the asymptotic formula in Theorem 1.5 matches the main term in [41] exactly, and one can see after some Gauss sums manipulations that the secondary main term in the asymptotic also matches the one obtained by Müller. The special case $\chi _1 = \chi$ and $\chi _2 = \overline{\chi }$ has been considered by Aryan [3], who extends the range to $h \ll X^{1-\epsilon }$ in this case using spectral methods.

### Remark 1.7

When $\chi _1 = \chi _2 = \chi$ is a quadratic character and $q \equiv 1 \hspace{0.05em}(\textrm{mod}\,4)$, $r_\chi$ is a norm-counting function for a quadratic extension of $\mathbb {F}_q[T]$ (see Sect. 2.2 for the precise definition). This is analogous to Müller’s motivating problem in [41] and is connected to moments of Dedekind zeta functions of quadratic extensions; see, for example, [31, 37, 40, 49].

The following theorem provides an asymptotic formula in the case $\ell _1 \ne \ell _2$.

### Theorem 1.8

Suppose that *h* is a polynomial with $\deg (h)=m$ and let $d =\max \{m,n\}$. Assume moreover that if $m=n$, then ${{\,\textrm{sgn}\,}}(h)\not =-1$. Let $\chi _1, \chi _2$ be primitive characters modulo $\ell _1$ and $\ell _2$ respectively, where $\ell _1 \ne \ell _2$. Further assume that $\ell _1, \ell _2 \ne 1$. Then

$$\begin{aligned}&\sum _{f \in \mathcal {M}_n} r_{\chi _1}(f) r_{\chi _2}(f+h) = \frac{ q^n L(1,\chi _1)L(1,\chi _2)}{L(2,\chi _1\chi _2)} \sum _{\begin{array}{c} g|h \\ \deg (g) \leqslant [d/2] \end{array}} \frac{\chi _1 \chi _2(g)}{|g|} \\&+ \frac{q^n \chi _2(\ell _1) L(1,\overline{\chi _1}) L(1,\chi _2) }{|\ell _1|L(2,\overline{\chi }_1\chi _2)} \sum _{\begin{array}{c} g|h \\ \deg (g) \leqslant [d/2]-\deg (\ell _1) \end{array}} \frac{\chi _2(g) \chi _1(h/g)}{|g_4|} \\&+\psi _{\chi _2}^\circ (h) \frac{q^n \chi _1(\ell _2) \overline{\chi _2}(-1) L(1,\chi _1) L(1,\overline{\chi _2}) }{|\ell _2|L(2,\chi _1 \overline{\chi _2})} \sum _{\begin{array}{c} g|h \\ \deg (g) \leqslant [(d-1)/2] \end{array}} \frac{\chi _1(g) \chi _2(h/g)}{|g|} \\&+\psi _{\chi _1,\chi _2}^{*}(h)\frac{q^n \overline{\chi _1}(\ell _2) \overline{\chi _2}(-\ell _1) L(1,\overline{\chi }_1)L(1,\overline{\chi _2})}{|\ell _1 \ell _2 | L(2,\overline{\chi _1 \chi _2}) } \sum _{\begin{array}{c} g|h \\ \deg (g) \leqslant [(d-1)/2] - \deg (\ell _1) \end{array}} \frac{ \chi _1 \chi _2(h/g)}{|g|} +E, \end{aligned}$$

where

$$ E \ll {\left\{ \begin{array}{ll}q^{3n/4+\epsilon n} &  \text{ if } m \leqslant n, n \equiv 0 \hspace{0.05em}(\textrm{mod}\,2), \deg (\ell )=1,\\ q^{3 d/4+ \deg (\ell _1)/2+ 3 \deg (\ell _2)/2+\epsilon d} &  \text{ otherwise, } \end{array}\right. } $$

and

$$\psi _{\chi _2}^\circ (h)={\left\{ \begin{array}{ll} \chi _2({{\,\textrm{sgn}\,}}(h))\overline{\chi _2}({{\,\textrm{sgn}\,}}(h)+1) &  \text{ if } m=n,\\ 1 &  \text{ if } m\not =n. \end{array}\right. }$$

We finally have the following two companion theorems.

### Theorem 1.9

Suppose that *h* is a polynomial with $\deg (h)=m$ and let $d =\max \{m,n\}$. Assume moreover that if $m=n$, then ${{\,\textrm{sgn}\,}}(h)\not =-1$. Let $\chi$ be a primitive character modulo $\ell$, where $\ell \ne 1$. Then

$$\begin{aligned} \sum _{f \in \mathcal {M}_n} d(f) r_{\chi }(f+h) = \frac{q^n L(1,\chi )}{L(2,\chi )} \sum _{\begin{array}{c} g|h \\ \deg (g) \leqslant [n/2] \end{array}} \frac{\chi (g)}{|g|} \Big [ n+1-2\deg (g)- \frac{2\,L'(2,\chi )}{(\log q) L(2,\chi )}\Big ]\\ +\psi _{\chi }^\circ (h)\frac{q^n \overline{\chi }(-1) L(1,\overline{\chi })}{|\ell |L(2,\overline{\chi })} \sum _{\begin{array}{c} g|h \\ \deg (g) \leqslant [n/2]-\deg (\ell ) \end{array}} \frac{\chi (h/g) }{|g|} \Big [n+1-2\deg (g \ell ) - \frac{2\,L'(2,\overline{\chi })}{(\log q) L(2,\overline{\chi })} \Big ]+ E, \end{aligned}$$

where

$$ E \ll {\left\{ \begin{array}{ll} q^{n/2+\epsilon n} &  \text{ if } m \leqslant n, \deg (\ell )=1 \text { or } m\! =\! n+1, n \text { odd }, \deg (\ell )\!=\!1, \\ q^{3d/4+3\deg (\ell )/2+\epsilon d} &  \text{ otherwise. } \end{array}\right. } $$

### Theorem 1.10

Suppose that *h* is a polynomial with $\deg (h)=m$ and let $d =\max \{m,n\}$. Assume moreover that if $m=n$, then ${{\,\textrm{sgn}\,}}(h)\not =-1$. Let $\chi$ is a primitive character modulo $\ell$, where $\ell \ne 1$. Then

$$\begin{aligned}  &   \sum _{f \in \mathcal {M}_n} r_{\chi }(f) d(f+h) = \frac{q^n L(1,\chi )}{L(2,\chi )} \sum _{\begin{array}{c} g|h \\ \deg (g) \leqslant [d/2] \end{array}} \frac{\chi (g)}{|g|} \Big [ d+1 - 2\deg (g)-\frac{2\,L'(2,\chi )}{(\log q) L(2,\chi )}\Big ]\\  &   +\frac{q^n L(1,\overline{\chi })}{|\ell |L(2,\overline{\chi })} \sum _{\begin{array}{c} g | h \\ \deg (g) \leqslant [d/2]-\deg (\ell ) \end{array}} \frac{ \chi (h/g)}{|g|} \Big [d+1-2\deg (g\ell ) - \frac{2\,L'(2,\overline{\chi })}{(\log q) L(2,\overline{\chi })} \Big ] +E, \end{aligned}$$

where

$$ E \ll {\left\{ \begin{array}{ll} q^{n/2+\epsilon n} &  \text{ if } m \leqslant n, \deg (\ell )=1, \\ q^{3d/4+\deg (\ell )/2+\epsilon d} &  \text{ otherwise. } \end{array}\right. } $$

Theorems 1.9 and 1.10 coincide when $\deg (h)\leqslant n$ after making the appropriate change of variables.

### Remark 1.11

The only analogue of Theorems 1.8, 1.9, and 1.10 in the integer setting (with or without smoothing) that we are aware of is an old work of Motohashi [38] using the dispersion method, where he studies the shifted convolution of *r*(*n*) and *d*(*n*). Since our results rely only on the Weil bound and not on spectral theory, we see no obstructions to translating our theorems to $\mathbb {Z}$.

Let us now discuss the proof of Theorem 1.1. We follow the strategy of Estermann: we open the divisor function $d(f+h)$ with a divisor switching trick to restrict to divisors $g \mid f+h$ satisfying $\deg (g) \leqslant d/2$. Thus, after swapping the order of summation, we need to understand

$$\begin{aligned} \sum _{\begin{array}{c} f \in \mathcal {M}_n \\ f \equiv -h \hspace{0.05em}(\textrm{mod}\,g) \end{array}} d(f) \text { for } \deg (g) \leqslant d/2. \end{aligned}$$

One may now use additive characters to detect the congruence. As usual, the zero frequency gives a dominant contribution and the goal is to bound the nonzero frequencies

5 $$\begin{aligned} \sum _{f \in \mathcal {M}_n} d(f)e\bigg (\frac{af}{g}\bigg ) \text { for }\deg (a) < \deg (g) \leqslant d/2 \text { and } (a,g) = 1. \end{aligned}$$

Here $e(\cdot )$ is the additive character defined in Sect. 2.1. It is now natural to look for a Voronoi-type summation formula for (5), which was hitherto unavailable. The main new contribution in this work is the formulation and derivation of such formulae (see Sects. 3–4). These results depend on a functional equation (Proposition 3.2) for (the function field analogue of) the Hurwitz zeta function. This formula will transform (5) into

$$\begin{aligned} \sum _{f \in \mathcal {M}_{\mu }} d(f) e\bigg (\frac{\overline{a} f}{g}\bigg ), \end{aligned}$$

with $\mu \approx d - n$ and $\overline{a}$ is the inverse of *a* modulo *g*. This is a beneficial move if $d < 2n$ which is why the error term in Theorem 1.1 only dominates the main term in this case. The sum over *a* yields a Kloosterman sum over $\mathbb {F}_q[T]$ (see (15)) and ultimately our theorem relies on the Linnik–Selberg conjecture for these sums, which was proved by Cogdell and Piatetski-Shapiro [16] in the function field setting. We note that many of the proofs of the analogous divisor correlation problem in the integer setting use considerable spectral input, as the Linnik–Selberg conjecture in this case is still open (see [45] for some recent progress and state of the art results for this problem).

The ranges in Theorem 1.8 are smaller than in Theorem 1.1 for two reasons. First, the conductor calculation implicit in the dual length being $\mu \approx d-n$ is no longer valid, since $\ell$ will also contribute to the conductor. Second, and more importantly, we do not have access to Linnik–Selberg type cancellation when the Kloosterman sums are twisted by multiplicative characters (even at a conjectural level, as far as we are aware). Sardari and Zargar [44] formulate a version of the conjecture when the twist is an additive character instead along with some divisibility conditions. This is wide open and the dependency on the twist and the divisibility conditions (which is essential for our application) is not explicit (see also the comment following the bound (18)). We must instead rely on a point-wise Weil bound [5, Lemma A.13] for these sums.

## Preliminaries and notation

We adopt the standard convention that $\epsilon$ is a sufficiently small quantity that may vary from line to line. We shall use asymptotic notation, such as $\ll ,\gg , O(\cdot ),$ and $o(\cdot )$ with their usual meanings. The implicit constants will usually depend on *q* (the size of the constant field) or $\epsilon$ but will be uniform in all other parameters.

### Basics of function fields

Let *p* be the characteristic of the function field so that $q = p^\eta$ for some $\eta \geqslant 1$. The set $\mathcal {M}= \cup _{n\geqslant 0} \mathcal {M}_n$ denotes the set of monic polynomials in $\mathbb {F}_q[T]$, with $\mathcal {M}_n$ being the ones of fixed degree *n*.

The only valuation of $\mathbb {F}_q(T)$ that we shall use will be the one at the infinite place, so we reserve the notation $|\cdot |$ for the associated norm. Thus,

6 $$\begin{aligned} |f| = q^{\deg f} = |\mathbb {F}_q[T]/(f)|, \end{aligned}$$

where the lattermost quantity is the cardinality of the residue ring. The completion of $\mathbb {F}_q(T)$ with respect to this norm is the ring of Laurent series in 1/*T*,

7 $$\begin{aligned} \mathbb {F}_q(T)_\infty = \bigg \{ \alpha = \sum _{j=-\infty }^N \alpha _j T^{j} : \alpha _j \in \mathbb {F}_q\text { and } N \in \mathbb {Z}\text { with } \alpha _N \ne 0 \bigg \}. \end{aligned}$$

We now fix an additive character on this ring. Denote by $e_p:\mathbb {F}_p \rightarrow \mathbb {C}^\times$ and $e_q:\mathbb {F}_q\rightarrow \mathbb {C}^\times$ the canonical additive characters,

$$\begin{aligned} e_p(x) = \exp \Big (\frac{2\pi i x}{p}\Big ), \qquad e_q(y) = e_p({{\,\textrm{Tr}\,}}(y)), \end{aligned}$$

where in the former $x \in \mathbb {F}_p$ and one interprets *x*/*p* as an element of $\mathbb {R}/\mathbb {Z}$ and in the latter $y \in \mathbb {F}_q$ and ${{\,\textrm{Tr}\,}}:\mathbb {F}_q\rightarrow \mathbb {F}_p$ is the (absolute) trace. The standard additive character on (7) is then defined by

$$\begin{aligned} e(\alpha ) = e_q({{\,\textrm{res}\,}}(\alpha )) = e_q(\alpha _{-1}), \end{aligned}$$

where $\alpha \in \mathbb {F}_q(T)_\infty$ and ${{\,\textrm{res}\,}}(\alpha )$ is the residue of $\alpha$ (i.e., the coefficient $\alpha _{-1}$).

For a primitive Dirichlet character $\chi$ modulo $\ell$, we recall from (4), $r_\chi = 1 \star \chi$ for the Dirichlet convolution

$$\begin{aligned} r_\chi (f) = \sum _{g \mid f} \chi (g). \end{aligned}$$

Here, and throughout, sums of the form $g \mid f$ run only over monic divisors $g \in \mathcal {M}$.

For *P* a monic irreducible polynomial, we also define the Legendre symbol by

8 $$\begin{aligned} \Big ( \frac{f}{P} \Big ) = f^{ \frac{|P|-1}{2}} \hspace{0.05em}(\textrm{mod}\,P), \end{aligned}$$

for $f \in \mathbb {F}_q[t]$. Note that $(\frac{f}{P}) \in \{ 0, \pm 1\}$.

For $k \geqslant 1$, $d_k(\cdot )$ is the generalized divisor function, and for $c \in \mathbb {F}_q[T]$, $g \in \mathcal {M}$, and $\Re (s) > 1$ we define

$$\begin{aligned} \zeta (s;c,g) = \sum _{\begin{array}{c} f \in \mathcal {M}\\ f \equiv c \hspace{0.05em}(\textrm{mod}\,g) \end{array}} \frac{1}{|f|^s}, \end{aligned}$$

and

$$\begin{aligned} D_k(s;c/g) = \sum _{f \in \mathcal {M}} \frac{d_k(f)e(cf/g)}{|f|^s}. \end{aligned}$$

These are the respective analogues of the Hurwitz zeta function and the (degree *k*) Estermann functions in $\mathbb {F}_q[T]$. Here the degree *k* indicates that we are working with the *k*-fold generalized divisor function, naturally associated to the *k*th power of the Riemann zeta function. In this article, we will mainly work with the cases $k=1,2$. The standard Estermann function over the integers was introduced by Estermann [26] who proved its meromorphic continuation and functional equation. Since then, the Estermann function has been used in a variety of other contexts (see, for example, [11, 12, 18, 27, 32, 39, 52]).

Throughout, we will let $u = q^{-s}$. Thus, $|f|^{-s} = u^{\deg f}$. With this convention, one may view the above as functions of *u*. When we do so, we will use script letters $\mathcal {Z}$ and $\mathcal {D}$ to emphasize the change-of-variables. That is, for $|u|<1/q$,

9 $$\begin{aligned} \mathcal {Z}(u;c,g) = \sum _{\begin{array}{c} f \in \mathcal {M}\\ f \equiv c \hspace{0.05em}(\textrm{mod}\,g) \end{array}} u^{\deg f}, \qquad \qquad \mathcal {D}_k(u;c/g) = \sum _{f \in \mathcal {M}} d_k(f)e(cf/g) u^{\deg (f)}. \end{aligned}$$

#### Remark 2.1

Observe that if $c \equiv c' \hspace{0.05em}(\textrm{mod}\,g)$, then

$$\mathcal {Z}(u;c,g) = \mathcal {Z}(u;c',g) \text { and } \mathcal {D}_1(u;c/g) = \mathcal {D}_1(u;c'/g).$$

A similar caveat applies to all other generating functions of this type such as $\mathcal {D}_2$ or $\mathcal {R}_2$ (defined below).

We define

$$\begin{aligned} \mathcal {R}_1\Big (u;\chi ,\frac{c}{g}\Big ):= \sum _{f \in \mathcal {M}} \chi (f) e\Big (\frac{cf}{g}\Big ) u^{\deg f}, \end{aligned}$$

$$\begin{aligned} \mathcal {Z}_\chi (u;c,g):= \sum _{\begin{array}{c} f \in \mathcal {M}\\ f \equiv c \hspace{0.05em}(\textrm{mod}\,g) \end{array}} \chi (f) u^{\deg f}, \end{aligned}$$

10 $$\begin{aligned} \mathcal {R}_2\Big (u;\chi , \frac{c}{g}\Big ) := \sum _{f \in \mathcal {M}} r_\chi (f) e\Big (\frac{cf}{g}\Big ) u^{\deg f}, \end{aligned}$$

which are the character twists of $\mathcal {D}_1$, $\mathcal {Z}$ and $\mathcal {D}_2$, respectively.

We also have the usual (Riemann) zeta function over $\mathbb {F}_q[T]$,

11 $$\begin{aligned} \zeta _{\mathbb {F}_q[T]}(s) = \sum _{f\in \mathcal {M}} \frac{1}{|f|^s}, \qquad \qquad \mathcal {Z}(u) = \sum _{f \in \mathcal {M}} u^{\deg f} = \frac{1}{1-qu}, \end{aligned}$$

the functional equation of which can be written as

12 $$\begin{aligned} \mathcal {Z}(u) = \frac{1-u}{u(qu-1)} \mathcal {Z}\Big (\frac{1}{qu}\Big ). \end{aligned}$$

For a Dirichlet character modulo ${\ell }$, we also define the *L*–function as

$$\begin{aligned} \mathcal {L}(u,\chi ) = \sum _{f \in \mathcal {M}} \chi (f) u^{\deg (f)}= \prod _P (1-\chi (P)u^{\deg (P)})^{-1}, \end{aligned}$$

where the product above is over monic, irreducible polynomials in $\mathbb {F}_q[t]$. When $\chi$ is a non-principal character modulo $\ell$, then $\mathcal {L}(u,\chi )$ is a polynomial of degree at most $\deg (\ell )-1$.

We will frequently use the Lindelöf bound for the *L*–function as follows. If $\chi$ is a non-principal character modulo $\ell$, for $|u| \leqslant q^{-1/2}$ and any $\epsilon >0$, we have that

13 $$\begin{aligned} |\mathcal {L}(u,\chi )| \ll |\ell |^{\epsilon }. \end{aligned}$$

(For a reference on this, see [20, Lemma 2.5] or [13, Theorem 5.1].) We shall also use the fact that for $|u| \leqslant 1/q$, we have

$$\begin{aligned} |\mathcal {L}(u,\chi )|^{-1} \ll |\ell |^{\epsilon }. \end{aligned}$$

For a proof of this, see Lemma 2.6 in [20].

Throughout the paper we will also frequently use Perron’s formula in function fields. Namely, if the power series $\mathcal {A}(u) = \sum _{f \in \mathcal {M}} a(f) u^{\deg (f)}$ is absolutely convergent in $|u| \leqslant r<1$, then

14 $$\begin{aligned} \sum _{f \in \mathcal {M}_n} a(f) = \frac{1}{2 \pi i} \oint _{|u|=r} \frac{\mathcal {A}(u)}{u^{n+1}} \, du, \, \, \, \, \, \sum _{f \in \mathcal {M}_{\leqslant n}} a(f) = \frac{1}{2 \pi i} \oint _{|u|=r} \frac{\mathcal {A}(u)}{u^{n+1}(1-u)} \, du. \end{aligned}$$

### Norm-counting functions for quadratic extensions

Let $\mathbb {A}$ be a Dedekind domain whose field of fractions *k* is a global field. Further, let *K* be a finite extension of *k* and let $\mathbb {B}$ be the integral closure of $\mathbb {A}$ in *K*. For an ideal $\mathfrak {b}$ in $\mathbb {B}$ (resp. an ideal $\mathfrak {a}$ in $\mathbb {A}$), one has the notion of absolute norm $|\cdot |$ given by

$$\begin{aligned} |\mathfrak {b}| = |\mathbb {B}/\mathfrak {b}| \qquad \text { (resp. } |\mathfrak {a}| = |\mathbb {A}/\mathfrak {a}|\text {)}, \end{aligned}$$

On the other hand, there is the notion of relative norm $N_{\mathbb {B}/\mathbb {A}}(\mathfrak {b})$ which is an ideal in $\mathbb {A}$ defined on primes $\mathfrak {P}$ by

$$\begin{aligned} N_{\mathbb {B}/\mathbb {A}}(\mathfrak {P}) = \mathfrak {p}^f, \end{aligned}$$

where $\mathfrak {p}= \mathfrak {P}\cap \mathbb {A}$ is the prime under $\mathfrak {P}$ and *f* is the residual (or inertial) degree of $\mathfrak {P}$. On other ideals of $\mathbb {B}$, $N_{\mathbb {B}/\mathbb {A}}$ is defined by complete multiplicativity. See [46, Chapter I, §5].

#### Remark 2.2

It is not difficult to check that the following diagram commutes, where *I* is the set of ideals.

We now take $\mathbb {A}= \mathbb {F}_q[T]$ so that $k = \mathbb {F}_q(T)$. All ideals $\mathfrak {a}= (f)$ are principal and the absolute norm agrees with the valuation of *f* as given in (6). We will also drop the subscript from $N_{\mathbb {B}/\mathbb {A}}$. The Dedekind zeta function of $\mathbb {B}$ is given by

$$\begin{aligned} \zeta _{\mathbb {B}}(s) = \sum _{\mathfrak {b}\subset \mathbb {B}} |\mathfrak {b}|^{-s}, \end{aligned}$$

where the sum runs over non-zero integral ideals of $\mathbb {B}$. This differs from the zeta function of *K* as a curve only in that it is missing the Euler factors corresponding to the infinite places of *K*. By Remark 2.2, we have that

$$\begin{aligned} \zeta _{\mathbb {B}}(s) = \sum _{\mathfrak {a}\subset \mathbb {A}} \frac{r_K(\mathfrak {a})}{|\mathfrak {a}|^{s}} = \sum _{f \in \mathcal {M}} \frac{r_K(f)}{|f|^s}, \end{aligned}$$

where the first sum runs over integral ideals of $\mathbb {A}$ and $r_K$ is the norm-counting function given by

$$\begin{aligned} r_K(\mathfrak {a}) = \#\{ \mathfrak {b}\subset \mathbb {B}: N(\mathfrak {b}) = \mathfrak {a}\}, \qquad \qquad r_K(f) = r_K((f)). \end{aligned}$$

Some basic properties of $r_K$ are proved in [9, §5.2].

We now assume that $q \equiv 1 \hspace{0.05em}(\textrm{mod}\,4)$ and assume that *K* is a quadratic extension of $\mathbb {F}_q(T)$ whose field of constants has not changed. Then, $\mathbb {B}= \mathbb {F}_q[T,\sqrt{\ell (T)}]$ for some squarefree $\ell \in \mathcal {M}_{\geqslant 1}$. Artin proved in his thesis [2, 42, Chapter 3] that, in this case,

$$\begin{aligned} \zeta _\mathbb {B}(s) = \zeta _{\mathbb {F}_q[T]}(s)L(s,\chi ) \end{aligned}$$

where $\chi$ is the primitive quadratic character given by the Kronecker symbol $\chi (f) = \Big ( \frac{\ell }{f} \Big )$, by quadratic reciprocity ([43, Theorem 3.3]) and (8). From this, it follows immediately that

$$\begin{aligned} r_K(f) = r_\chi (f). \end{aligned}$$

See also Remark 1.7.

### Character sums over $\mathbb {F}_q[T]$

For $f,h \in \mathbb {F}_q[T]$ and $g \in \mathcal {M}$, we denote the Kloosterman sum over $\mathbb {F}_q[T]$ by15where the $*$ indicates that the sum runs over coprime residue classes modulo *g*, and $\overline{a}$ is the multiplicative inverse of *a* modulo *g*.

For $\ell \in \mathcal {M}$ and a character $\chi$ modulo $\ell$, we denote the Gauss sum by

16 $$\begin{aligned} G(\chi ,f) = \sum _{a \hspace{0.05em}(\textrm{mod}\,\ell )} \chi (a)e\Big (\frac{af}{\ell }\Big ), \qquad G(\chi ) = G(\chi ,1). \end{aligned}$$

Note that if $\chi$ is primitive then $G(\chi ,f) = \overline{\chi }(f) G(\chi )$.

Throughout the paper, we will use the following point-wise bound for the Kloosterman sum. Lemma $\textrm{A}.13$ in [5] gives that

17 $$\begin{aligned} |S(f,h;g)| \leqslant 2^{\omega (g)} q^{\frac{\deg (g)}{2}+ \frac{ \deg (f,h,g)}{2}}, \end{aligned}$$

where $\omega (g)$ denotes the number of monic, irreducible factors of *g*.

We will also use the Linnik–Selberg bound for averages of Kloosterman sums over function fields (see Remark 3 following Conjecture 1.4 in [44] and [16]). Namely,

18 $$\begin{aligned} \Big | \sum _{g \in \mathcal {M}_n} S(f,h;g) \Big | \ll q^{n(1+\epsilon )} |fh|^{\epsilon }. \end{aligned}$$

Note that in [44], the authors formulate a more general version of the conjecture than the bound above, summing over $g \in \mathcal {M}_n$ satisfying certain divisibility conditions, as well as twisting by an additive character. As mentioned before, this twisted version is wide open, and moreover, the dependency on the divisibility parameter and the twist is not explicit.

### Character sums over $\mathbb {F}_q$

The orthogonality of additive characters states that

$$\begin{aligned} 1\!\!1_{x = 0} = \frac{1}{q} \sum _{\lambda \in \mathbb {F}_q} e_q(\lambda x) \end{aligned}$$

for $x \in \mathbb {F}_q$. Separating the term $\lambda = 0$ and rearranging, we have that

19 $$\begin{aligned} \sum _{\lambda \in \mathbb {F}_q^\times } e_q(\lambda x) = - 1 + q1\!\!1_{x = 0}, \end{aligned}$$

which will be useful in Sect. 3.

The *r*-dimensional hyper-Kloosterman sum over $\mathbb {F}_q$ will be denoted by ${{\,\textrm{Kl}\,}}_r$. For $\alpha _1,\cdots ,\alpha _r \in \mathbb {F}_q$, this is defined by

$$\begin{aligned} {{\,\textrm{Kl}\,}}_r(\alpha _1,\cdots ,\alpha _r) = \sum _{\begin{array}{c} \gamma _1,\cdots ,\gamma _n \in \mathbb {F}_q^\times \\ \gamma _1\gamma _2 \cdots \gamma _n = 1 \end{array}} e_q(\alpha _1 \gamma _1 + \alpha _2 \gamma _2 + \cdots +\alpha _n \gamma _n). \end{aligned}$$

The important cases for us are

20 $$\begin{aligned} {{\,\textrm{Kl}\,}}_1(\lambda ) = e_q(\lambda ), \qquad {{\,\textrm{Kl}\,}}_2(\alpha ,\beta ) = \sum _{\gamma \in \mathbb {F}_q^\times }e_q(\alpha \gamma + \beta \gamma ^{-1}). \end{aligned}$$

Further, it is not hard to see that

21 $$\begin{aligned} {{\,\textrm{Kl}\,}}_2(\alpha ,0) = {{\,\textrm{Kl}\,}}_2(0,\alpha ) = -1 \text { when } \alpha \ne 0 \end{aligned}$$

and

22 $$\begin{aligned} {{\,\textrm{Kl}\,}}_2(0,0) = q-1. \end{aligned}$$

For a multiplicative character of $\mathbb {F}_q$, $\chi$, we will also need the $\chi$-twisted Kloosterman sum ${{\,\textrm{Kl}\,}}_{\chi }$ defined by

23 $$\begin{aligned} {{\,\textrm{Kl}\,}}_{\chi }(\alpha ,\beta ) = \sum _{\gamma \in \mathbb {F}_q^\times } \chi (\gamma ) e_q(\alpha \gamma + \beta \gamma ^{-1}). \end{aligned}$$

#### Remark 2.3

If $\chi$ is a Dirichlet character over $\mathbb {F}_q[T]$, then the restriction $\chi \big |_{\mathbb {F}_q^\times }$ is a bonafide multiplicative character of $\mathbb {F}_q$ and hence has an associated twisted Kloosterman sum. We will slightly abuse notation and write ${{\,\textrm{Kl}\,}}_\chi$ also for the latter.

An important special case of (23) is

24 $$\begin{aligned} {{\,\textrm{Kl}\,}}_\chi (\alpha ,0) = \sum _{\gamma \in \mathbb {F}_q^\times } \chi (\gamma ) e_q(\alpha \gamma ) =: \tau (\chi ,\alpha ), \end{aligned}$$

which is the Gauss sum of (the restriction of) $\chi$ over $\mathbb {F}_q$. Similarly,

25 $$\begin{aligned} {{\,\textrm{Kl}\,}}_\chi (0,\beta ) = \sum _{\gamma \in \mathbb {F}_q^\times } \chi (\gamma )e_q(\beta \gamma ^{-1}) = \tau (\overline{\chi },\beta ),\end{aligned}$$

by substituting $\gamma$ by $\gamma ^{-1}$ and noting that $\overline{\chi }(\gamma ) = \chi (\gamma ^{-1})$. Finally,

26 $$\begin{aligned} {{\,\textrm{Kl}\,}}_\chi (0,0) = \sum _{\gamma \in \mathbb {F}_q^\times } \chi (\gamma ) = (q-1)1\!\!1(\chi \text { is even}),\end{aligned}$$

where we recall that $\chi$ is an even Dirichlet character if it is trivial on the units $\mathbb {F}_q^\times$ and is odd otherwise.

We also set,

$$\begin{aligned} \tau (\chi ) = \tau (\chi ,1) = \sum _{\gamma \in \mathbb {F}_q^\times } \chi (\gamma ) e_q(\gamma ). \end{aligned}$$

## Functional equations

Recall the definition (9) of $\mathcal {Z}(u;c,g)$ and $\mathcal {D}_k(u;c/g)$, which holds for $|u|<1/q$.

### Degree one

Our first goal is to derive functional equations for the degree one functions $\mathcal {Z}$ and $\mathcal {D}_1$. We have the following representation for these functions, which implicitly gives us a meromorphic continuation to the whole complex plane.

#### Lemma 3.1

Let $g \in \mathcal {M}$ and $c \in \mathbb {F}_q[T]$ with $\deg (c) < \deg (g)$. Then, for all $u \in \mathbb {C}$, we have that

27 $$\begin{aligned} \mathcal {Z}(u;c,g) = \frac{u^{\deg (g)}}{1 - qu}+ 1\!\!1_{c\in \mathcal {M}} u^{\deg (c)}. \end{aligned}$$

If, further, $c \ne 0$, then we have that

28 $$\begin{aligned} \mathcal {D}_{1}\Big (u; \frac{c}{g} \Big ) = (qu)^{\deg (g) - \deg (c) - 1}\bigg ( e_q\big ({{\,\textrm{sgn}\,}}(c)\big ) - \frac{1}{1 - qu} \bigg ) + \frac{1}{1 - qu}, \end{aligned}$$

where $1\!\!1_{c\in \mathcal {M}}$ is an indicator function detecting whether *c* is monic and ${{\,\textrm{sgn}\,}}(c)$ is the leading coefficient of *c* as a polynomial.

#### Proof

By the principle of analytic continuation, we may assume that $|u|<1/q$ for the rest of proof. To prove the first equality, we note that writing $f = g f_1 + c$, we find that since *g* is monic and $\deg (c)<\deg (g)$, then the condition that $f \in \mathcal {M}$ is equivalent to $f_1 \in \mathcal {M}$ *unless* *c* is monic and $f_1 = 0$. In the former case, $\deg (f) = \deg (gf_1 + c) = \deg (g) + \deg (f_1)$, whence $|f| = |g||f_1|$. In the latter case, $|f|=|c|$. Thus, we find that

$$\begin{aligned} \zeta (s;c,g) = \sum _{\begin{array}{c} f \in \mathcal {M}\\ f\equiv c \hspace{0.05em}(\textrm{mod}\,g) \end{array}} |f|^{-s} = |g|^{-s} \sum _{f_1 \in \mathcal {M}} |f_1|^{-s} + 1\!\!1_{c\in \mathcal {M}} |c|^{-s}. \end{aligned}$$

The claim now follows from (11) by rewriting both sides as functions of $u = q^{-s}$. Note that this argument also applies for $g = 1$ (in which case *c* is necessarily 0).

To prove the second equality, we first note (by Taylor expansion) that the right hand side is a polynomial in *u*, given by

$$\begin{aligned} \sum _{n = 0}^{\deg (g)-\deg (c) - 1} a_n u^n, \end{aligned}$$

with $a_n = q^n$ for $0 \leqslant n \leqslant \deg (g) - \deg (c) - 2$ and $a_n = q^n e_q\big ({{\,\textrm{sgn}\,}}(c)\big )$ for $n = \deg (g) - \deg (c) - 1$. On the other hand, arranging the left hand side by the degree of *f*, we find that

$$\begin{aligned} \mathcal {D}_1(u;c/g) = \sum _{f \in \mathcal {M}} e\Big (\frac{cf}{g}\Big ) u^{\deg f} = \sum _{n=0}^\infty u^n \sum _{f \in \mathcal {M}_n} e\Big (\frac{cf}{g}\Big ). \end{aligned}$$

The equality then follows from the fact [30, Theorem 3.7] that under the hypotheses of the lemma,

$$\begin{aligned} \frac{1}{q^n}\sum _{f\in \mathcal {M}_n} e\Big (\frac{cf}{g}\Big ) = {\left\{ \begin{array}{ll} \;\; 1 &  \text {if }n < \deg (g) - \deg (c) -1, \\ \;\; e_q\big ({{\,\textrm{sgn}\,}}(c)\big ) &  \text {if }n = \deg (g) - \deg (c) - 1, \\ \;\; 0 &  \text {if }n > \deg (g) - \deg (c) - 1. \end{array}\right. } \end{aligned}$$

Note that in this case, we need not worry about the case $g = 1$, since that is incompatible with the requirements that $c \ne 0$ and $\deg (c) < \deg (g)$. $\square$

It is worth remarking that the above lemma implies that

$$\begin{aligned} \mathcal {Z}(u;0,g) = u^{\deg (g)} \mathcal {Z}(u), \end{aligned}$$

with $\mathcal {Z}(u)$ given in (11). On the other hand, it follows directly from the definition that

$$\begin{aligned} \mathcal {D}_1(u;0) = \mathcal {Z}(u). \end{aligned}$$

We are now ready to prove the functional equation of the Hurwitz zeta function.

#### Proposition 3.2

(Functional equation of $\mathcal {Z}$) Let $g \in \mathcal {M}$ and $c \in \mathbb {F}_q[T]$. Then, for all $u \in \mathbb {C}$, we have that

29 $$\begin{aligned} \mathcal {Z}(u;c,g)= u^{\deg (g)-1} \sum _{\lambda \in \mathbb {F}_q^\times } \bigg [ \frac{e_q(-\lambda )}{q}-\frac{1}{q(1-qu)} \bigg ] \mathcal {D}_1\Big ( \frac{1}{qu}, \frac{\lambda c}{g}\Big ). \end{aligned}$$

We remark that the above equality is still true for $u=\frac{1}{q}$ if we interpret it as saying that both sides have simple poles with the same residue.

#### Proof

First, since both sides depend only on the congruence class of *c* modulo *g* by Remark 2.1, we can assume without loss of generality that $\deg (c) < \deg (g)$.

Let us quickly dispose of the case $c = 0$ by noting that in this case $\mathcal {Z}(u;0,g)$ on the left hand side becomes $u^{\deg (g)} \mathcal {Z}(u)$ while every copy of $\mathcal {D}_1$ on the right hand side of (29) is equal to $\mathcal {Z}(1/qu)$. Thus, we may execute the sum over $\lambda$ by using (19). On doing so, (29) simply becomes the functional equation of the Riemann zeta function over $\mathbb {F}_q[t]$, (12), multiplied by $u^{\deg (g)}$.

When $c \ne 0$, we can replace *u* by 1/(*qu*) in (28) and simplify, to find that

$$\begin{aligned} \mathcal {D}_1\Big (\frac{1}{qu}; \frac{\lambda c}{g}\Big ) = u^{\deg (c) + 1 - \deg (g)}\bigg ( e_q\big ({{\,\textrm{sgn}\,}}(\lambda c)\big ) - \frac{u}{u - 1} \bigg ) + \frac{u}{u - 1}. \end{aligned}$$

Since ${{\,\textrm{sgn}\,}}(\lambda c) = \lambda {{\,\textrm{sgn}\,}}(c)$, we see that the right hand side of (29) may be written as

30 $$\begin{aligned} \sum _{\lambda \in \mathbb {F}_q^\times } \Big [A e_q(\lambda ({{\,\textrm{sgn}\,}}(c) - 1)) + B e_q(\lambda {{\,\textrm{sgn}\,}}(c)) + C e_q(-\lambda ) + D\Big ], \end{aligned}$$

where

$$\begin{aligned} A: = \frac{u^{\deg (c)}}{q}, \qquad B:= -\frac{u^{\deg (c)}}{q(1-qu)}, \end{aligned}$$

$$\begin{aligned} C:= \frac{u^{\deg (g)} - u^{\deg (c) + 1}}{q(u-1)}, \qquad D:= \frac{u^{\deg (c) + 1}-u^{\deg (g)}}{q(1-qu)(u-1)}. \end{aligned}$$

By orthogonality (cf. (19)), (30) is equal to

$$\begin{aligned} - A + qA 1\!\!1_{{{\,\textrm{sgn}\,}}(c) - 1 = 0} - B - C + (q-1)D. \end{aligned}$$

Now, by a routine calculation, we find that

$$\begin{aligned} -A - B - C + (q-1)D = \frac{u^{\deg (g)}}{1-qu}, \end{aligned}$$

while clearly,

$$\begin{aligned} qA1\!\!1_{{{\,\textrm{sgn}\,}}(c) - 1 = 0} = 1\!\!1_{c \in \mathcal {M}} u^{\deg (c)}. \end{aligned}$$

Note that, by (27), the sum of the above two expressions is precisely the left hand side of (29), completing the proof. $\square$

#### Remark 3.3

The expression in square parentheses in (29) may be written as

$$\begin{aligned} \sum _{\eta \in \{0,1\}} (-1)^{\eta } \frac{e_q\big ((\eta -1)\lambda \big )}{q(1-qu)^{\eta }} = \sum _{\eta \in \{0,1\}} (-1)^{\eta } \frac{{{\,\textrm{Kl}\,}}_1\big ((\eta -1)\lambda \big )}{q(1-qu)^{\eta }}. \end{aligned}$$

This will be useful later.

Proposition 3.2 can be reversed (for example, by basic linear algebra over $\mathbb {C}(u)$) to obtain the functional equation of $\mathcal {D}_1$. It is slightly more convenient to proceed directly, with a similar argument.

#### Proposition 3.4

(Functional equation of $\mathcal {D}_1$) Let $g \in \mathcal {M}$ and $c \in \mathbb {F}_q[T]$. Then, for all $u \in \mathbb {C}$, we have that

31 $$\begin{aligned} \mathcal {D}_{1}\Big (u; \frac{c}{g} \Big ) = (qu)^{\deg (g)-1} \sum _{\lambda \in \mathbb {F}_q^\times } \bigg [e_q(\lambda ^{-1})-\frac{1}{1-qu}\bigg ] \mathcal {Z}\Big (\frac{1}{qu};\lambda c,g\Big ). \end{aligned}$$

We remark that the above equality is still true for $g \mid c$ and $u=\frac{1}{q}$ if we interpret it as saying that both sides have simple poles with the same residue. Note that both sides have no poles if $g\not \mid c$.

#### Proof

We proceed similarly to the proof of Proposition 3.2. As before, we may restrict without losing generality to $\deg (c) < \deg (g)$ with $c \ne 0$ (in the case $c = 0$, (31) devolves to (12) by applying (19)) and thus, by (27),

$$\begin{aligned} \mathcal {Z}\Big (\frac{1}{qu};\lambda c, g\Big ) = (qu)^{-\deg (g)}\frac{u}{u - 1}+ 1\!\!1_{\lambda c\in \mathcal {M}} (qu)^{-\deg (c)}, \end{aligned}$$

whence the right hand side of (31) may be written as

32 $$\begin{aligned} \sum _{\lambda \in \mathbb {F}_q^\times } \Big [A' e_q(\lambda ^{-1})1\!\!1_{\lambda c \in \mathcal {M}} + B'1\!\!1_{\lambda c \in \mathcal {M}} + C' e_q(\lambda ^{-1}) + D'\Big ], \end{aligned}$$

with

$$\begin{aligned} A': = (qu)^{\deg (g) - \deg (c) - 1}, \qquad B':= -\frac{(qu)^{\deg (g) - \deg (c)-1}}{1-qu}, \end{aligned}$$

$$\begin{aligned} C':= \frac{1}{q(u-1)}, \qquad D':= -\frac{1}{q(1-qu)(u-1)}. \end{aligned}$$

Note that $\lambda \rightarrow \lambda ^{-1}$ is an involution on $\mathbb {F}_q^\times$, so that (32) is equal to

$$\begin{aligned} {\begin{matrix} \sum _{\lambda \in \mathbb {F}_q^\times } \Big [A' e_q(\lambda )1\!\!1_{\lambda ^{-1} c \in \mathcal {M}} + B'1\!\!1_{\lambda ^{-1} c \in \mathcal {M}} + C' e_q(\lambda ) + D'\Big ] \\ = A'e_q\big ({{\,\textrm{sgn}\,}}(c)\big ) + B' -C' + (q-1)D', \end{matrix}} \end{aligned}$$

where we have used (19) together with the fact that $\lambda ^{-1} c \in \mathcal {M}$ if and only if $\lambda = {{\,\textrm{sgn}\,}}(c)$. The result now follows from observing that

$$\begin{aligned} A'e_q\big ({{\,\textrm{sgn}\,}}(c)\big ) + B' = (qu)^{\deg (g) - \deg (c) - 1}\bigg ( e_q\big ({{\,\textrm{sgn}\,}}(c)\big ) - \frac{1}{1 - qu} \bigg ) \end{aligned}$$

is the first term in (28) while

$$\begin{aligned} - C' + (q-1) D' = \frac{1}{1-qu} \end{aligned}$$

is the second. $\square$

#### Remark 3.5

Similar to Remark 3.3, the expression in square parentheses in (31) may be written as

$$\begin{aligned} \sum _{\eta \in \{0,1\}} (-1)^{\eta } \frac{e_q\big ((1-\eta )\lambda ^{-1}\big )}{(1-qu)^{\eta }} = \sum _{\eta \in \{0,1\}} (-1)^{\eta } \frac{{{\,\textrm{Kl}\,}}_1\big ((1-\eta )\lambda ^{-1}\big )}{(1-qu)^{\eta }}. \end{aligned}$$

### Degree two

We will now use Propositions 3.2 and 3.4 in tandem to prove the functional equation for $\mathcal {D}_2$. For this, we need a lemma that expresses $\mathcal {D}_2$ as a combination of $\mathcal {Z}$ and $\mathcal {D}_1$. This lemma also implicitly provides meromorphic continuation for $\mathcal {D}_2$ to the whole complex plane.

#### Lemma 3.6

Let $g \in \mathcal {M}$ and let $c \in \mathbb {F}_q[T]$. Then, for all $u \in \mathbb {C}$, we have that

33 $$\begin{aligned} \mathcal {D}_2\Big (u;\frac{c}{g}\Big ) = \sum _{a \hspace{0.05em}(\textrm{mod}\,g)} \mathcal {Z}(u;a,g)\mathcal {D}_1\Big (u;\frac{ac}{g}\Big ). \end{aligned}$$

If we further assume that $(c,g) = 1$ and let $b\in \mathbb {F}_q[T]$ (not necessarily coprime to *g*), then for all $u \in C$, we have that

34 $$\begin{aligned} \mathcal {D}_2\Big (u;\frac{b\overline{c}}{g}\Big ) = \sum _{a \hspace{0.05em}(\textrm{mod}\,g)} \mathcal {Z}(u;ac,g)\mathcal {D}_1\Big (u;\frac{ab}{g}\Big ), \end{aligned}$$

where $\overline{c}$ is any inverse of *c* modulo *g*.

We remark that the above equalities are still true for $u=\frac{1}{q}$ if we interpret them as saying that both sides have poles of order two with the same residue.

#### Proof

As in the proof of Lemma 3.1, we may assume $|u| < 1/q$ for absolute convergence. On doing so, we find that

35 $$\begin{aligned} \mathcal {D}_2\Big (u;\frac{c}{g}\Big ) = \sum _{f_1,f_2 \in \mathcal {M}} e\Big (\frac{cf_1f_2}{g}\Big ) u^{\deg (f_1) + \deg (f_2)}. \end{aligned}$$

We now collect the terms with $f_1$ in a particular congruence class *a* modulo *g* together. For all such terms, $e(cf_1f_2/g) = e(acf_2/g)$; from this it follows that

$$\begin{aligned} \mathcal {D}_2\Big (u;\frac{c}{g}\Big ) = \sum _{a \hspace{0.05em}(\textrm{mod}\,g)} \sum _{\begin{array}{c} f_1 \in \mathcal {M}\\ f_1 \equiv a \hspace{0.05em}(\textrm{mod}\,g) \end{array}} u^{\deg (f_1)} \sum _{f_2 \in \mathcal {M}} e\Big (\frac{acf_2}{g}\Big ) u^{\deg (f_2)}. \end{aligned}$$

We can close the sum over $f_2$ as a copy of $\mathcal {D}_1(u;ac/g)$, while the sum over $f_1$ will then yield a copy of $\mathcal {Z}(u;a,g)$; from this we obtain

$$\begin{aligned} \mathcal {D}_2\Big (u;\frac{c}{g}\Big ) = \sum _{a \hspace{0.05em}(\textrm{mod}\,g)} \mathcal {Z}(u;a,g)\mathcal {D}_1\Big (u;\frac{ac}{g}\Big ), \end{aligned}$$

as desired to prove (33).

To prove (34), we first replace *c* with $b\overline{c}$ and *a* with $a_1$ in (33) to get

$$\begin{aligned} \mathcal {D}_2\Big (u;\frac{b\overline{c}}{g}\Big ) = \sum _{a_1 \hspace{0.05em}(\textrm{mod}\,g)} \mathcal {Z}(u;a_1,g)\mathcal {D}_1\Big (u;\frac{a_1b\overline{c}}{g}\Big ). \end{aligned}$$

Now, we make the substitution $a_1= ac$. This gives

$$\begin{aligned} \mathcal {D}_2\Big (u;\frac{b\overline{c}}{g}\Big ) = \sum _{a \hspace{0.05em}(\textrm{mod}\,g)} \mathcal {Z}(u;ac,g)\mathcal {D}_1\Big (u;\frac{acb\overline{c}}{g}\Big ) = \sum _{a \hspace{0.05em}(\textrm{mod}\,g)} \mathcal {Z}(u;ac,g)\mathcal {D}_1\Big (u;\frac{ab}{g}\Big ), \end{aligned}$$

as desired. Here, we have used $c\overline{c} \equiv 1 \hspace{0.05em}(\textrm{mod}\,g)$, together with Remark 2.1. $\square$

We are now in a position to prove the functional equation for the Estermann function, $\mathcal {D}_2$.

#### Proposition 3.7

Let $g \in \mathcal {M}_{\geqslant 1}$ and $c\in \mathbb {F}_q[T]$ be such that $(c,g) = 1$. Then, for all $u \in \mathbb {C}$, we have that

36 $$\begin{aligned} \mathcal {D}_2\Big (u;\frac{c}{g}\Big ) = (qu^2)^{\deg (g) -1} \sum _{\lambda \in \mathbb {F}_q^\times } \mathcal {A}_\lambda (u) \mathcal {D}_2\Big (\frac{1}{qu};\frac{\lambda \overline{c}}{g}\Big ), \end{aligned}$$

where $\overline{c}$ is any inverse of *c* modulo *g* and

37 $$\begin{aligned} \begin{aligned} \mathcal {A}_\lambda (u) :&= \sum _{\eta _1,\eta _2 \in \{0,1\}}\bigg [ (-1)^{\eta _1+\eta _2}\frac{{{\,\textrm{Kl}\,}}_2\big (\eta _1-1,(1-\eta _2)\lambda \big )}{q (1-qu)^{\eta _1 + \eta _2}}\bigg ] \\  &= \frac{{{\,\textrm{Kl}\,}}_2(-1,\lambda )}{q} + \frac{1+q - 2qu}{q(1-qu)^2}. \end{aligned} \end{aligned}$$

Here, ${{\,\textrm{Kl}\,}}_2$ is as in (20).

We remark that equality (36) is still true for $u=\frac{1}{q}$ if we interpret it as saying that both sides have poles of order two with the same residue.

The reader may wish to compare (36) to the functional equation of the Estermann function in the integer setting (e.g. (19) in [26]), which relates the function *D*(*s*, *a*/*q*) (for coprime integers *a* and *q*) to $D(1-s,\overline{a}/q)$ and $D(1-s,-\overline{a}/q)$. In that case, there are two terms indexed by $\{\pm \}$ in $\mathbb {Z}$; in contrast, here there are $q-1$ terms indexed by $\lambda \in \mathbb {F}_q^\times$. This may be surprising at first sight, but there is a natural reason: the indexing set is precisely the units in the corresponding rings, namely $\{\pm \}$ in $\mathbb {Z}$ and $\mathbb {F}_q^\times$ in $\mathbb {F}_q[T]$.

#### Proof of Proposition 3.7

Before proving (36), let us establish the second equality in (37). By (21) and (22), we have

$$\begin{aligned} \begin{aligned} \mathcal {A}_\lambda (u)&= \frac{{{\,\textrm{Kl}\,}}_2(-1,\lambda )}{q} - \frac{{{\,\textrm{Kl}\,}}_2(-1,0)}{q(1-qu)} - \frac{{{\,\textrm{Kl}\,}}_2(0,\lambda )}{q(1-qu)} + \frac{{{\,\textrm{Kl}\,}}_2(0,0)}{q(1-qu)^2} \\  &= \frac{{{\,\textrm{Kl}\,}}_2(-1,\lambda )}{q} + \frac{1}{q(1-qu)} + \frac{1}{q(1-qu)} + \frac{q-1}{q(1-qu)^2}. \end{aligned} \end{aligned}$$

The claim follows on combining the latter three terms above.

The proof strategy for (36) is hopefully clear: we use Lemma 3.6 to write $\mathcal {D}_2$ as a combination of $\mathcal {D}_1$ and $\mathcal {Z}$ and apply the functional equations (cf. Propositions 3.4 and 3.2) of $\mathcal {D}_1$ and $\mathcal {Z}$. To put this into effect, we substitute (29) and (31) into the right hand side of (33)—with *c* replaced by *a* or *ac*, as necessary—to find that

38 $$\begin{aligned} \mathcal {D}_2\Big (u;\frac{c}{g}\Big )= &   (qu^2)^{\deg (g)-1}\sum _{a \hspace{0.05em}(\textrm{mod}\,g)}\left( \sum _{\lambda _1 \in \mathbb {F}_q^\times }\mathcal {D}_1\Big (\frac{1}{qu};\frac{\lambda _1 a}{g}\Big ) \sum _{\eta _1 \in \{0,1\}} (-1)^{\eta _1} \frac{e_q\big ((\eta _1 - 1)\lambda _1\big )}{q(1-qu)^{\eta _1}} \right. \nonumber \\    &   \qquad \quad \left. \times \sum _{\lambda _2 \in \mathbb {F}_q^\times } \mathcal {Z}\Big (\frac{1}{qu};\lambda _2ac,g\Big ) \sum _{\eta _2 \in \{0,1\}} (-1)^{\eta _2} \frac{e_q\big ((1-\eta _2)\lambda _2^{-1}\big )}{(1-qu)^{\eta _2}} \right) , \end{aligned}$$

where we have implicitly used Remarks 3.3 and 3.5.

We now focus only on the sum over *a*. We have

$$\begin{aligned} \sum _{a\hspace{0.05em}(\textrm{mod}\,g)} \mathcal {Z}\Big (\frac{1}{qu};\lambda _2 ac,g\Big ) \mathcal {D}_1\Big (\frac{1}{qu};\frac{\lambda _1 a}{g}\Big ) = \mathcal {D}_2\Big (\frac{1}{qu};\frac{\lambda _1\lambda _2^{-1}\overline{c}}{g}\Big ), \end{aligned}$$

where we have used (34) after making the appropriate substitutions. Substituting this back into (38) and rearranging, we see that

$$\begin{aligned} \mathcal {D}_2\Big (u;\frac{c}{g}\Big )= &   (qu^2)^{\deg (g)-1}\sum _{\lambda _1,\lambda _2 \in \mathbb {F}_q^\times } \Bigg (\sum _{\eta _1,\eta _2 \in \{0,1\}} (-1)^{\eta _1+\eta _2} \\  &   \quad \quad \times \frac{e_q\big ((\eta _1 - 1)\lambda _1+(1-\eta _2)\lambda _2^{-1}\big )}{q(1-qu)^{\eta _1+\eta _2}} \mathcal {D}_2\Big (\frac{1}{qu};\frac{\lambda _1\lambda _2^{-1}\overline{c}}{g}\Big )\Bigg ). \end{aligned}$$

We now collect the terms which have the same sign $\lambda = \lambda _1\lambda _2^{-1}$ together. Thus, the sum over $(\lambda _1,\lambda _2)$ may be replaced by a sum over $(\lambda ,\lambda _1)$. On doing so, and rearranging with the sum over $\lambda$ outside, and the sum over $\lambda _1$ all the way inside, we get

$$\begin{aligned} \mathcal {D}_2\Big (u;\frac{c}{g}\Big ) = (qu^2)^{\deg (g)-1}\sum _{\lambda \in \mathbb {F}_q^\times } \mathcal {D}_2\Big (\frac{1}{qu};\frac{\lambda \overline{c}}{g}\Big ) \bigg [\sum _{\eta _1,\eta _2 \in \{0,1\}} \frac{(-1)^{\eta _1+\eta _2}}{q(1-qu)^{\eta _1+\eta _2}} \\ \times \sum _{\lambda _1\in \mathbb {F}_q^\times } e_q\big ((\eta _1 - 1)\lambda _1+(1-\eta _2)\lambda \lambda _1^{-1}\big )\bigg ]. \end{aligned}$$

We can now close the sum over $\lambda _1$ as ${{\,\textrm{Kl}\,}}_2(\eta _1 - 1,(1-\eta _2)\lambda )$, whence (36) follows by noting that the quantity in the square brackets in the right hand side is precisely $\mathcal {A}_\lambda (u)$. $\square$

#### Remark 3.8

The identity

$$\begin{aligned} \mathcal {D}_2\Big (u;\frac{c}{g}\Big ) = \sum _{a,b \hspace{0.05em}(\textrm{mod}\,g)} e\Big (\frac{abc}{g}\Big ) \mathcal {Z}(u;a,g)\mathcal {Z}(u;b,g) \end{aligned}$$

holds simply by fibering the right hand side of (35) in terms of congruences classes of both $f_1$ and $f_2$ modulo *g*. With this as a starting point, an alternate proof of Proposition 3.7 may be obtained by applying the functional equation of $\mathcal {Z}$ twice. In the classical setting, this is essentially Estermann’s original strategy [24] (see also [17, Lemma 4]). This route is marginally tedious compared to our approach (especially for character-twisted analogues and the degree 3 analogue à la Fazzari [27]; see Sect. 3.3 and the remark below), so we leave the details to the interested reader.

#### Remark 3.9

An analogue of Propositions 3.4 and 3.7 for the *GL*(3) Estermann function, $\mathcal {D}_3(u;c/g)$, has been obtained by the authors, the details of which will appear elsewhere.

### Character twists

In this section, $\chi$ is a fixed primitive character modulo $\ell$. We are making neither the assumption that $\ell$ is prime nor the assumption that $\ell = 1$ here, though one of these assumptions will become necessary in later sections. Our goal is to prove a functional equation for $\mathcal {R}_2(u;\chi ,c/g)$, given in (10). This is more complicated when the greatest common divisor $(\ell ,g) \notin \{1,\ell \}$, so we restrict ourselves to the cases $(\ell ,g) = 1$ and $(\ell ,g) = \ell$. The reader interested in the general case may try adapting [49, Theorem 2.3].

#### Lemma 3.10

Let $g,\ell \in \mathcal {M}$ and $c \in \mathbb {F}_q[T]$ such that $(\ell ,g) = 1$. Further, let $\chi$ be a primitive Dirichlet character modulo $\ell$. Then, for all $u \in \mathbb {C}$, we have that

39 $$\begin{aligned} \mathcal {R}_1\Big (u;\chi ,\frac{c}{g}\Big )= &   \chi (g)G(\chi )(qu)^{\deg (g)-1} u^{\deg (\ell )} \nonumber \\  &   \qquad \quad \times \sum _{\lambda \in \mathbb {F}_q^\times } \chi (-\lambda )\bigg [e_q(\lambda ^{-1}) - \frac{1}{1-qu}\bigg ] \mathcal {Z}_{\overline{\chi }}\Big (\frac{1}{qu};\lambda c\ell , g\Big ), \end{aligned}$$

where $G(\chi )$ is the Gauss sum of $\chi$ defined in (16).

Here there are no poles except in the case where $\ell = 1$ and $g\mid c$, which reduces to Proposition 3.4.

#### Proof

From the definition of $\mathcal {R}_1$, it follows that

$$\begin{aligned} \mathcal {R}_1\Big (u;\chi ,\frac{c}{g}\Big ) = \sum _{a_1 \hspace{0.05em}(\textrm{mod}\,\ell )} \sum _{a_2\hspace{0.05em}(\textrm{mod}\,g)} \chi (a_1) e\Big (\frac{a_2c}{g}\Big ) \sum _{\begin{array}{c} f \equiv a_1 \hspace{0.05em}(\textrm{mod}\,\ell )\\ f \equiv a_2 \hspace{0.05em}(\textrm{mod}\,g) \end{array}} u^{\deg f}, \end{aligned}$$

by fibering the sum over $f \in \mathcal {M}$ according to the congruence class of *f* modulo *g* (say $a_1$) and $\ell$ (say $a_2$) respectively.

Now, let $\overline{g}$ represent any fixed multiplicative inverse of *g* modulo $\ell$ (and the same for $\overline{\ell }$ with roles reversed). These are well-defined as $(\ell ,g) = 1$. By the Chinese Remainder Theorem, if we set

40 $$\begin{aligned} a : = a_1 g\overline{g} + a_2 \ell \overline{\ell }, \end{aligned}$$

where *a* depends on $a_1$ and $a_2$, then the congruence conditions in the right-most sum above may be replaced by $f \equiv a \hspace{0.05em}(\textrm{mod}\,g\ell )$. Doing so and recalling the definition of $\mathcal {Z}$ in (9) yields the identity

$$\begin{aligned} \mathcal {R}_1\Big (u;\chi ,\frac{c}{g}\Big ) = \sum _{a_1 \hspace{0.05em}(\textrm{mod}\,\ell )} \sum _{a_2\hspace{0.05em}(\textrm{mod}\,g)} \chi (a_1) e\Big (\frac{a_2c}{g}\Big ) \mathcal {Z}(u;a,g\ell ). \end{aligned}$$

Inserting the functional equation of $\mathcal {Z}$ above (cf. (29)), we find the following expression for $\mathcal {R}_1(u;\chi ,c/g)$:

$$\begin{aligned} \sum _{a_1 \hspace{0.05em}(\textrm{mod}\,\ell )} \sum _{a_2\hspace{0.05em}(\textrm{mod}\,g)} \chi (a_1) e\bigg (\frac{a_2c}{g}\bigg )\! \sum _{\lambda \in \mathbb {F}_q^\times } \bigg [ \frac{e_q(\lambda ^{-1})}{q} - \frac{1}{q(1-qu)}\bigg ]u^{\deg (g\ell )-1} \mathcal {D}_1\Big (\frac{1}{qu},\frac{\lambda a}{g\ell }\Big ). \end{aligned}$$

We now open the definition of $\mathcal {D}_1$ as a sum over *f* and push the sums over $a_1$ and $a_2$ inside. Recalling the definition of *a* from (40) and focusing only on the terms depending on $a_1$ and $a_2$, we find

$$\begin{aligned} \sum _{a_1 \hspace{0.05em}(\textrm{mod}\,\ell )} \sum _{a_2\hspace{0.05em}(\textrm{mod}\,g)} \chi (a_1) e\bigg (\frac{a_2c}{g} + \frac{\lambda a_2\overline{\ell } f}{g} + \frac{\lambda a_1 \overline{g} f}{\ell }\bigg ). \end{aligned}$$

We can perform the summation over $a_2$ using orthogonality of characters. This yields the congruence condition $f \equiv - c\lambda ^{-1} \ell \hspace{0.05em}(\textrm{mod}\,g)$ and contributes a factor of $|g| = q^{\deg (g)}$. On the other hand, closing the sum over $a_1$ yields a Gauss sum

$$\begin{aligned} G(\chi ,\lambda \overline{g} f) = \chi (\lambda ^{-1})\chi (g)\overline{\chi }(f) G(\chi ), \end{aligned}$$

where we have used the primitivity of $\chi$. From here, we get that

$$\begin{aligned} \mathcal {R}_1\Big (u;\chi ,\frac{c}{g}\Big )= &   \chi (g)G(\chi )(qu)^{\deg (g)-1} u^{\deg (\ell )} \sum _{\lambda \in \mathbb {F}_q^{\times }} \chi (\lambda ^{-1})\bigg [e_q(-\lambda ) - \frac{1}{1-qu}\bigg ] \\  &   \qquad \qquad \quad \times \sum _{\begin{array}{c} f \in \mathcal {M}\\ f \equiv -\lambda ^{-1}c\ell \hspace{0.05em}(\textrm{mod}\,g) \end{array}} \overline{\chi }(f) (qu)^{-\deg f}. \end{aligned}$$

Here it is worth remarking that we have consolidated the powers of *q* arising from the congruence sums with the extra factor inside the $\lambda$-sum. Making the change of variables $\lambda \mapsto -\lambda ^{-1}$ and closing the sum over *f*, we obtain

$$\begin{aligned} \mathcal {R}_1\Big (u;\chi ,\frac{c}{g}\Big )= &   \chi (g)G(\chi )(qu)^{\deg (g)-1} u^{\deg (\ell )} \\    &   \quad \quad \times \sum _{\lambda \in \mathbb {F}_q^\times } \chi (-\lambda )\bigg [e_q(\lambda ^{-1}) - \frac{1}{1-qu}\bigg ] \mathcal {Z}_{\overline{\chi }}\Big (\frac{1}{qu};\lambda c\ell , g\Big ), \end{aligned}$$

as desired. $$\square $$

#### Remark 3.11

It is worth noting that the term in square brackets in (39) is the same as in Remark 3.5 and so may be written as

$$\begin{aligned} \sum _{\eta \in \{0,1\}} (-1)^{\eta } \frac{e_q\big ((1-\eta )\lambda ^{-1}\big )}{(1-qu)^{\eta }}. \end{aligned}$$

The case $g = 1$ of the above lemma is particularly interesting, since it gives an alternate proof of the functional equation for a primitive Dirichlet *L*-function, $\mathcal {L}(u,\chi )$, which is similar to Hurwitz’s original proof of the functional equation in the integer setting (see the discussion in Davenport [19, pp. 65, 71–72]).

The stage is now set for proving a functional equation for $\mathcal {R}_2(u;\chi ,c/g)$ when $(\ell ,g) = 1$.

#### Proposition 3.12

Let $g \in \mathcal {M}_{\geqslant 1}$, $\ell \in \mathcal {M}$, and $c\in \mathbb {F}_q[T]$ be such that $(\ell c,g) = 1$. Further, let $\chi$ be a primitive Dirichlet character modulo $\ell$. Then, for all $u \in \mathbb {C}$, we have that

41 $$\begin{aligned} \mathcal {R}_2\Big (u;\chi ,\frac{c}{g}\Big ) =\chi (g)G(\chi )(qu^2)^{\deg (g)-1} u^{\deg (\ell )} \sum _{\lambda \in \mathbb {F}_q^\times } \mathcal {B}_{\lambda }(u;\overline{\chi })\mathcal {R}_2\Big (\frac{1}{qu};\overline{\chi },\frac{\lambda \overline{c}}{g}\Big ), \end{aligned}$$

where

42 $$\begin{aligned} \begin{aligned} \mathcal {B}_\lambda (u;\chi )&:= \sum _{\eta _1,\eta _2 \in \{0,1\}} (-1)^{\eta _1+\eta _2}\frac{{{\,\textrm{Kl}\,}}_{\chi }\big (\eta _1-1,(1-\eta _2)\lambda \big )}{q(1-qu)^{\eta _1+\eta _2}} \\  &= \frac{{{\,\textrm{Kl}\,}}_{\chi }(-1,\lambda )}{q} - \bigg [\frac{\tau (\chi ,-1) + \tau (\overline{\chi },\lambda )}{q(1 - qu)}\bigg ] + \frac{q-1}{q(1-qu)^2} 1\!\!1(\chi is even ). \end{aligned} \end{aligned}$$

Here, $G(\chi )$, ${{\,\textrm{Kl}\,}}_\chi$, $\tau (\chi ,\cdot )$ are as in (16), (23), (24).

We remark that equality (41) is still true for $u=\frac{1}{q}$ if we interpret it as saying that both sides have simple poles with the same residue except in the case $\ell = 1$ where we reduce to Proposition 3.7.

#### Proof

For $(\ell c,g) = 1$ and any $b \in \mathbb {F}_q[T]$, the identities

43 $$\begin{aligned} \mathcal {R}_2\Big (u;\chi ,\frac{c}{g}\Big ) = \sum _{a \hspace{0.05em}(\textrm{mod}\,g)} \mathcal {Z}(u;a,g) \mathcal {R}_1\Big (u;\chi ,\frac{ac}{g}\Big ) \end{aligned}$$

and

44 $$\begin{aligned} \mathcal {R}_2\Big (u;\chi , \frac{b\overline{c}}{g}\Big ) = \sum _{a \hspace{0.05em}(\textrm{mod}\,g)} \mathcal {Z}_\chi (u;ac,g) \mathcal {D}_1\Big (u,\frac{ab}{g}\Big ) \end{aligned}$$

can be shown to hold entirely analogously to Lemma 3.6. Substituting the functional equations for $\mathcal {Z}$ and $\mathcal {R}_1$ (i.e., (29) and (39)) into the right hand side of (43), we get

$$\begin{aligned}  &   \mathcal {R}_2\Big (u;\chi , \frac{c}{g}\Big ) = \chi (g)G(\chi )(qu^2)^{\deg (g)-1} u^{\deg (\ell )} \\  &   \quad \times \sum _{a\hspace{0.05em}(\textrm{mod}\,g)}\Bigg (\sum _{\lambda _2 \in \mathbb {F}_q^\times } \bigg [\sum _{\eta _2 \in \{0,1\}} (-1)^{\eta _2} \frac{e_q\big ((\eta _2-1)\lambda _2\big )}{q(1-qu)^{\eta _2}}\bigg ] \mathcal {D}_1\Big (\frac{1}{qu};\frac{\lambda _2 a}{g}\Big ) \\  &   \quad \times \sum _{\lambda _1 \in \mathbb {F}_q^\times }\chi (-\lambda _1)\bigg [\sum _{\eta _1\in \{0,1\}} (-1)^{\eta _1} \frac{e_q\big ((1-\eta _1)\lambda _1^{-1}\big )}{(1-qu)^{\eta _1}}\bigg ] \mathcal {Z}_{\overline{\chi }}\Big (\frac{1}{qu};\lambda _1ac,g\Big ) \Bigg ). \end{aligned}$$

Here, we have incorporated Remarks 3.3 and  3.11. Using (44), we can execute the sum over $a \hspace{0.05em}(\textrm{mod}\,g)$ to get

$$\begin{aligned}  &   \mathcal {R}_2\Big (u;\chi , \frac{c}{g}\Big ) = \chi (g)G(\chi )(qu^2)^{\deg (g)-1} u^{\deg (\ell )} \sum _{\lambda _1,\lambda _2 \in \mathbb {F}_q^\times } \chi (-\lambda _1) \\    &   \times \bigg [\sum _{\eta _1,\eta _2 \in \{0,1\}} (-1)^{\eta _1+\eta _2} \frac{e_q\big ((1-\eta _1)\lambda _1^{-1}+(\eta _2-1)\lambda _2\big )}{q(1-qu)^{\eta _1+\eta _2}}\bigg ] \mathcal {R}_2\Big (\frac{1}{qu};\overline{\chi },\frac{\lambda _1^{-1}\lambda _2\overline{c}}{g}\Big ). \end{aligned}$$

Letting $\lambda = \lambda _1^{-1} \lambda _2$, writing the sums over $(\lambda _1,\lambda _2)$ as a sum over $(\lambda ,\lambda _1)$, and pushing the sum over $\lambda _1$ inside we find that the right hand side above transforms into

$$\begin{aligned} \chi (g)G(\chi )(qu^2)^{\deg (g)-1} u^{\deg (\ell )} \sum _{\lambda \in \mathbb {F}_q^\times } \mathcal {R}_2\Big (\frac{1}{qu};\overline{\chi },\frac{\lambda \overline{c}}{g}\Big ) \bigg [\sum _{\eta _1,\eta _2 \in \{0,1\}} \frac{(-1)^{\eta _1+\eta _2}}{q(1-qu)^{\eta _1+\eta _2}}\bigg ] \\ \times \sum _{\lambda _1 \in \mathbb {F}_q^\times } \chi (-\lambda _1) e_q\big ((1-\eta _1)\lambda _1^{-1}+(\eta _2-1)\lambda \lambda _1\big ). \end{aligned}$$

To finish the proof of (41), we need to show that the sum over $\lambda _1$ above is equal to

$$\begin{aligned} {{\,\textrm{Kl}\,}}_{\overline{\chi }}\big (\eta _1-1,(1-\eta _2)\lambda \big ) = \sum _{\gamma \in \mathbb {F}_q^\times } \overline{\chi }(\gamma ) e_q\big ((\eta _1-1)\gamma + (1-\eta _2)\lambda \gamma ^{-1}\big ), \end{aligned}$$

which can be seen by substituting $\lambda _1 = -\gamma ^{-1}$ and by noting that $\chi (\gamma ^{-1}) = \overline{\chi }(\gamma )$; in this way, we find that the sum over $\eta _1,\eta _2$ in the above expression gives $\mathcal {B}_\lambda (u;\chi )$ as desired.

It remains to show the second equality in (42). Thus,

$$\begin{aligned} \begin{aligned} \mathcal {B}_\lambda (u;\chi )&= \frac{{{\,\textrm{Kl}\,}}_\chi (-1,\lambda )}{q} - \frac{{{\,\textrm{Kl}\,}}_\chi (-1,0)}{q(1-qu)} - \frac{{{\,\textrm{Kl}\,}}_\chi (0,\lambda )}{q(1-qu)} + \frac{{{\,\textrm{Kl}\,}}_{\chi }(0,0)}{q(1-qu)^2} \\&= \frac{{{\,\textrm{Kl}\,}}_\chi (-1,\lambda )}{q} - \frac{\tau (\chi ,-1)}{q(1-qu)} - \frac{\tau (\overline{\chi },\lambda )}{q(1-qu)} + \frac{q-1}{q(1-qu)^2}1\!\!1(\chi \text { is even}), \end{aligned} \end{aligned}$$

by (24), (25), and (26). $$\square $$

#### Remark 3.13

Notice that if $\chi$ is even, then $\tau (\chi ,\lambda ) = -1$ for $\lambda \ne 0$. Thus, we find that whenever $\chi$ is even, $\mathcal {B}_\lambda (u;\chi ) = \mathcal {A}_\lambda (u)$ for every $\lambda \in \mathbb {F}_q^\times$.

#### Remark 3.14

The above proposition remains valid when $\ell = 1$. In this case, we see that there is only one primitive character modulo $\ell$ (namely, the constant function $f \mapsto 1$). Thus, $\mathcal {R}_2(u;1,c/g) = \mathcal {D}_2(u;c/g)$ and in view of the previous remark, Proposition 3.12 simply becomes Proposition 3.7.

We finish off this section by establishing a version of Proposition 3.12 in the alternate situation where $\ell$ divides *g*.

#### Proposition 3.15

Let $g \in \mathcal {M}_{\geqslant 1}$, $\ell \in \mathcal {M}$, and $c\in \mathbb {F}_q[T]$ be such that $\ell \mid g$ and $(c,g) = 1$. Further, let $\chi$ be a primitive Dirichlet character modulo $\ell$. Then, for all $u \in \mathbb {C}$, we have that

$$\begin{aligned} \mathcal {R}_2\Big (u;\chi ,\frac{c}{g}\Big ) = \overline{\chi }(-c) (qu^2)^{\deg (g) - 1} \sum _{\lambda \in \mathbb {F}_q^\times } \mathcal {B}_\lambda (u;\chi ) \mathcal {R}_2\Big (\frac{1}{qu};\chi ,\frac{\lambda \overline{c}}{g}\Big ), \end{aligned}$$

where $\mathcal {B}_\lambda (u;\chi )$ is the same as in (42).

We remark that equality (41) is still true for $u=\frac{1}{q}$ if we interpret it as saying that both sides have simple poles with the same residue except in the case $\ell = 1$ where we reduce to Proposition 3.7.

#### Proof

By opening $r_\chi (f) = \sum _{f_1 f_2 = f} \chi (f_2)$ and fibering the sum according to the congruence class of $f_2$ modulo *g*, one finds that

45 $$\begin{aligned} \mathcal {R}_2\Big (u;\chi ,\frac{c}{g}\Big ) = \sum _{a \hspace{0.05em}(\textrm{mod}\,g)} \chi (a) \mathcal {Z}(u;a,g)\mathcal {D}_1\Big (u;\frac{ac}{g}\Big ). \end{aligned}$$

Similarly, for $$(c,g) = 1$$,

46 $$\begin{aligned} \chi (\overline{c}) \mathcal {R}_2\Big (u;\chi ,\frac{b\overline{c}}{g}\Big ) = \sum _{a \hspace{0.05em}(\textrm{mod}\,g)} \chi (a) \mathcal {Z}(u;ac,g) \mathcal {D}_1\Big (u;\frac{ab}{g}\Big ). \end{aligned}$$

Here, $\overline{c}$ is the multiplicative inverse of *c* modulo *g* (and hence, also modulo $\ell$). These are analogous to (43) and (44), but depend greatly on the fact that $\ell \mid g$.

Applying the functional equations for $\mathcal {D}_1$ and $\mathcal {Z}$ (cf. Propositions 3.2 and 3.4), the right hand side of (45) becomes

$$\begin{aligned} (qu^2)^{\deg (g) - 1} \sum _{a\hspace{0.05em}(\textrm{mod}\,g)} \Bigg ( \chi (a) \sum _{\lambda _1,\lambda _2 \in \mathbb {F}_q^\times }\sum _{\eta _1,\eta _2 \in \{0,1\}} (-1)^{\eta _1+\eta _2} \frac{e_q\big ((1-\eta _1)\lambda _1^{-1}+(\eta _2-1)\lambda _2\big )}{q(1-qu)^{\eta _1+\eta _2}} \\ \times \mathcal {Z}\Big (\frac{1}{qu};\lambda _1 ac, g\Big )\mathcal {D}_1\Big (\frac{1}{qu};\frac{\lambda _2 a}{g}\Big ) \Bigg ). \end{aligned}$$

Executing the sum over $a \bmod g$ using (46), we get

$$\begin{aligned}  &   \mathcal {R}_2\Big (u;\chi ,\frac{c}{g}\Big ) = (qu^2)^{\deg (g) - 1} \sum _{\lambda _1,\lambda _2 \in \mathbb {F}_q^\times }\sum _{\eta _1,\eta _2 \in \{0,1\}} (-1)^{\eta _1+\eta _2} \\  &   \qquad \quad \times \frac{e_q\big ((1-\eta _1)\lambda _1^{-1}+(\eta _2-1)\lambda _2\big )}{q(1-qu)^{\eta _1+\eta _2}} \times \chi (\lambda _1^{-1} \overline{c}) \mathcal {R}_2\Big (\frac{1}{qu};\chi ,\frac{\lambda _1^{-1} \lambda _2\overline{c}}{g}\Big ). \end{aligned}$$

Now, we follow the usual strategy of letting $\lambda = \lambda _1^{-1}\lambda _2$ and replacing sums over $(\lambda _1,\lambda _2)$ by $(\lambda ,\lambda _1)$. In particular, we see that the sum over $\lambda _1$ is given by

$$\begin{aligned} \sum _{\lambda _1 \in \mathbb {F}_q^\times } \chi (\lambda _1^{-1}) e_q\big ((1-\eta _1)\lambda _1^{-1}+(\eta _2-1)\lambda \lambda _1\big ) = \chi (-1) {{\,\textrm{Kl}\,}}_\chi \big (\eta _1-1,(1-\eta _2)\lambda ), \end{aligned}$$

by making the substitution $\lambda _1 = -\gamma ^{-1}$ and recalling (23). Thus, we find

$$\begin{aligned} \mathcal {R}_2\Big (u;\chi ,\frac{c}{g}\Big ) = \overline{\chi }(-c) (qu^2)^{\deg (g) - 1} \sum _{\lambda \in Fq^\times } \mathcal {B}_\lambda (u;\chi ) \mathcal {R}_2\Big (\frac{1}{qu};\chi ,\frac{\lambda \overline{c}}{g}\Big ), \end{aligned}$$

as desired. $$\square $$

#### Remark 3.16

As with Proposition 3.12, one can set $\ell = 1$ in Proposition 3.15 in which case we get back Proposition 3.7.

## Voronoi summation formulae

In this section, we shall derive the requisite Voronoi summation formulae from the functional equations we found in Sect. 3. The reader may wish to compare the formulae to Theorems 4.13 and 4.14 in [35, Chapter 4].

### Proposition 4.1

Let $g \in \mathcal {M}_{\leqslant n/2}$, $\ell \in \mathcal {M}$, and $c \in \mathbb {F}_q[T]$ be such that $(\ell c,g) = 1$ and let $r_\chi$ be as in (4) with $\chi$ a primitive Dirichlet character modulo $\ell$. Then,

47 $$\begin{aligned}  &   \sum _{f \in \mathcal {M}_n} r_\chi (f)e\Big (\frac{cf}{g}\Big ) = \mathscr {M}_n\nonumber \\    &   \quad + \frac{q^n}{|g\ell |}\chi (g)G(\chi ) \sum _{\lambda \in \mathbb {F}_q^\times } \sum _{\eta _1,\eta _2 \in \{0,1\}} \sum _{0\leqslant k \leqslant \mu } b_{\mu -k}(\overline{\chi }) \sum _{f \in \mathcal {M}_k} r_{\overline{\chi }}(f)e\Big (\frac{\lambda \overline{c}f}{g}\Big ) \end{aligned}$$

where $$\mu = 2\deg (g) +\deg (\ell ) - n - 2$$,

48 $$\begin{aligned} \mathscr {M}_n = - {{\,\textrm{res}\,}}_{u = 1/q} \bigg (\frac{\mathcal {R}_2(u;\chi ,c/g)}{u^{n+1}}\bigg ), \end{aligned}$$

$b_k = b_k(\chi ;\lambda ,\eta _1,\eta _2)$ is given by

49 $$\begin{aligned} b_k(\chi ;\lambda ,\eta _1,\eta _2) = (-1)^{k-\eta _1-\eta _2}{{\,\textrm{Kl}\,}}_\chi \big (\eta _1-1,(1-\eta _2)\lambda \big ) \times \Big [1\!\!1_{k \geqslant \eta _1 + \eta _2} \left( {\begin{array}{c}-\eta _1 - \eta _2\\ k - \eta _1 - \eta _2\end{array}}\right) \Big ]. \end{aligned}$$

Notice that when $\mu < 0$, the second term in (47) is simply 0.

### Proof

Let

$$\begin{aligned} \mathscr {I}(\sigma ,n) = \frac{1}{2\pi i}\oint _{|u|=q^{-\sigma }} \mathcal {R}_2\Big (u;\chi ,\frac{c}{g}\Big ) \frac{du}{u^{n+1}}, \end{aligned}$$

where the contour $|u| = q^{-\sigma }$ is being traversed counter-clockwise. Note that the integrand has poles only at $u = 1/q$ (coming from $\mathcal {R}_2$) and $u = 0$, whence two applications of Cauchy’s residue theorem give that

$$\begin{aligned} \mathscr {I}(-1,n) = - \mathscr {M}_n + \mathscr {I}(2,n). \end{aligned}$$

On the other hand, Perron’s formula (14) tells us that

$$\begin{aligned} \mathscr {I}(2,n) = \sum _{f \in \mathcal {M}_n} r_\chi (f)e\Big (\frac{cf}{g}\Big ). \end{aligned}$$

Thus, it suffices to show that

$$\begin{aligned} \mathscr {I}(-1,n) = \frac{q^n}{|g\ell |}\chi (g)G(\chi ) \sum _{\lambda \in \mathbb {F}_q^\times } \sum _{\eta _1,\eta _2 \in \{0,1\}} \sum _{0\leqslant k \leqslant \mu } b_{\mu -k}(\overline{\chi }) \sum _{f \in \mathcal {M}_k} r_{\overline{\chi }}(f)e\Big (\frac{\lambda \overline{c}f}{g}\Big ). \end{aligned}$$

To do this, we apply Proposition 3.12 to the integrand of $\mathscr {I}(-1,n)$. On doing so, and pulling the sum over $\lambda$ out, we obtain that

$$\begin{aligned} \mathscr {I}(-1,n) = \frac{q^{\deg (g) - 1}\chi (g)G(\chi )}{2\pi i}\sum _{\lambda \in \mathbb {F}_q^\times } \oint _{|u|=q} \mathcal {B}_\lambda (u;\overline{\chi })\mathcal {R}_2\Big (\frac{1}{qu};\overline{\chi },\frac{\lambda \overline{c}}{g}\Big )\frac{du}{u^{n-2\deg (g)-\deg (\ell ) + 3}}. \end{aligned}$$

We now substitute $u = 1/(qv)$ and recall the definition of $\mu$, finding that

$$\begin{aligned} \mathscr {I}(-1,n)= &   q^{n - \deg (g) - \deg (\ell ) + 1}\chi (g)G(\chi ) \\  &   \qquad \quad \times \frac{1}{2\pi i} \sum _{\lambda \in \mathbb {F}_q^\times } \oint _{|v|=q^{-2}} \mathcal {B}_\lambda \Big (\frac{1}{qv};\overline{\chi }\Big )\mathcal {R}_2\Big (v;\overline{\chi },\frac{\lambda \overline{c}}{g}\Big )\frac{dv}{v^{\mu +1}}, \end{aligned}$$

where we have consolidated the factors of *q* coming from the change of variables together with the existing factor $q^{\deg (g) - 1}$. Now, by substituting $u = 1/(qv)$ in (42) and simplifying, we obtain

$$\begin{aligned} \begin{aligned} \mathcal {B}_\lambda \Big (\frac{1}{qv};\overline{\chi }\Big )&= \sum _{\eta _1,\eta _2 \in \{0,1\}} \frac{{{\,\textrm{Kl}\,}}_{\overline{\chi }}\big (\eta _1-1,(1-\eta _2)\lambda \big )}{q} \times \frac{v^{\eta _1 + \eta _2}}{(1-v)^{\eta _1 + \eta _2}} \\  &= \frac{1}{q}\sum _{\eta _1,\eta _2 \in \{0,1\}} \sum _{k=0}^\infty b_k (\overline{\chi }) v^k. \end{aligned} \end{aligned}$$

The last step follows from the binomial theorem. Substituting this above, we get

$$\begin{aligned} \mathscr {I}(-1,n) = \frac{q^n}{|g\ell |}\chi (g)G(\chi ) \sum _{\lambda \in \mathbb {F}_q^\times }\sum _{\eta _1,\eta _2\in \{0,1\}}\sum _{k=0}^\infty \frac{b_k(\overline{\chi }) }{2\pi i}\oint _{|v|=q^{-2}} \mathcal {R}_2\Big (v;\overline{\chi },\frac{\lambda \overline{c}}{g}\Big ) \frac{dv}{v^{\mu - k + 1}}. \end{aligned}$$

The inner integral vanishes if $k > \mu$, whence the sum over *k* can be truncated to $k \leqslant \mu$. Then, using Perron’s formula to evaluate the integral, we get

$$\begin{aligned} \mathscr {I}(-1,n) = \sum _{\lambda \in \mathbb {F}_q^\times }\sum _{\eta _1,\eta _2\in \{0,1\}}\sum _{0\leqslant k \leqslant \mu } b_k(\overline{\chi }) \bigg [\frac{q^n}{|g\ell |}\chi (g)G(\chi )\sum _{f\in \mathcal {M}_{\mu -k}} r_{\overline{\chi }}(f)e\Big (\frac{\lambda \overline{c}f}{g}\Big )\bigg ], \end{aligned}$$

completing the proof. $\square$

We will need an explicit evaluation of the term $\mathscr {M}_n$ in the above proposition.

### Lemma 4.2

Under the same assumptions as Proposition 4.1 and the additional assumption that $\chi$ is non-principal (equivalently, that $\ell \in \mathcal {M}_{\geqslant 1}$) we have that

$$\begin{aligned} \mathscr {M}_{n} = \frac{q^n}{|g|}\chi (g)L(1,\chi ), \end{aligned}$$

where $\mathscr {M}_n$ is as in (48) and $L(s,\chi )$ is the Dirichlet *L*-function associated to $\chi$.

### Proof

Collecting the terms $f_1, f_2$ according to their congruence classes *a* and *b* modulo *g* and applying (27), we obtain

50 $$\begin{aligned} \mathcal {R}_2\Big (u;\chi ,\frac{c}{g}\Big )&= \sum _{f_1, f_2 \in \mathcal {M}} \chi (f_1) e \Big ( \frac{cf_1f_2}{g} \Big ) u^{\deg (f_1)+\deg (f_2)} \nonumber \\&= \sum _{a, b \hspace{0.05em}(\textrm{mod}\,g)} e \Big ( \frac{abc}{g} \Big ) \mathcal {Z}_{\chi }(u;b,g) \mathcal {Z}(u;a,g)\nonumber \\&= \sum _{a, b \hspace{0.05em}(\textrm{mod}\,g)} e \Big ( \frac{abc}{g} \Big ) \mathcal {Z}_{\chi }(u;b,g) \Big [ \frac{u^{\deg (g)}}{1-qu}+ 1\!\!1_{a \in \mathcal {M}}u^{\deg (a)}\Big ]. \end{aligned}$$

If we now focus on the term $\mathcal {Z}_{\chi }(u;b,g)$ and again collect terms according to their congruences modulo $g \ell$, we have, by applying (27) again,

51 $$\begin{aligned} \mathcal {Z}_{\chi }(u;b,g)&= \sum _{\begin{array}{c} r \hspace{0.05em}(\textrm{mod}\,g\ell ) \\ r \equiv b \hspace{0.05em}(\textrm{mod}\,g) \end{array}} \chi (r) \sum _{f \equiv r \hspace{0.05em}(\textrm{mod}\,g \ell )} u^{\deg (f)}\nonumber \\&= \sum _{\begin{array}{c} r \hspace{0.05em}(\textrm{mod}\,g\ell ) \\ r \equiv b \hspace{0.05em}(\textrm{mod}\,g) \end{array}} \chi (r) \Big [ \frac{u^{\deg (g \ell )}}{1-qu} +1\!\!1_{r \in \mathcal {M}} u^{\deg (r)} \Big ]\nonumber \\&= \frac{u^{\deg (g \ell )}}{1-qu} \sum _{a \hspace{0.05em}(\textrm{mod}\,\ell )} \chi (a) \sum _{\begin{array}{c} r \hspace{0.05em}(\textrm{mod}\,g \ell )\\ r \equiv b \hspace{0.05em}(\textrm{mod}\,g) \\ r \equiv a \hspace{0.05em}(\textrm{mod}\,\ell ) \end{array}} 1 + \sum _{\begin{array}{c} r \hspace{0.05em}(\textrm{mod}\,g\ell ) \\ r \equiv b \hspace{0.05em}(\textrm{mod}\,g)\\ r \in \mathcal {M} \end{array}} \chi (r) u^{\deg (r)} \end{aligned}$$

52 $$\begin{aligned}&= \sum _{\begin{array}{c} r \hspace{0.05em}(\textrm{mod}\,g\ell ) \\ r \equiv b \hspace{0.05em}(\textrm{mod}\,g)\\ r \in \mathcal {M} \end{array}} \chi (r) u^{\deg (r)}. \end{aligned}$$

Note that in equation (51) we used the fact that there is a unique solution $r \hspace{0.05em}(\textrm{mod}\,g \ell )$ such that $r \equiv b \hspace{0.05em}(\textrm{mod}\,g)$ and $r \equiv a \hspace{0.05em}(\textrm{mod}\,\ell )$, since $(\ell ,g)=1$, which implies that the sum over *r* is equal to 1, and then the sum over $a \hspace{0.05em}(\textrm{mod}\,\ell )$ vanishes by orthogonality of characters. Replacing (52) in (50), dividing by $u^{n+1}$, and computing the residue at $u=1/q$ yields

$$\begin{aligned} \mathscr {M}_n = \frac{q^n}{|g|} \sum _{a,b \hspace{0.05em}(\textrm{mod}\,g)} e\Big ( \frac{abc}{g}\Big ) \sum _{\begin{array}{c} r \hspace{0.05em}(\textrm{mod}\,g\ell ) \\ r \equiv b \hspace{0.05em}(\textrm{mod}\,g)\\ r \in \mathcal {M} \end{array}} \frac{\chi (r)}{|r|}. \end{aligned}$$

It remains to evaluate the above sums. We have

$$\begin{aligned} \sum _{a,b \hspace{0.05em}(\textrm{mod}\,g)}&e\Big ( \frac{abc}{g}\Big ) \sum _{\begin{array}{c} r \hspace{0.05em}(\textrm{mod}\,g\ell ) \\ r \equiv b \hspace{0.05em}(\textrm{mod}\,g)\\ r \in \mathcal {M} \end{array}} \frac{\chi (r)}{|r|} = \sum _{\begin{array}{c} r \hspace{0.05em}(\textrm{mod}\,g \ell ) \\ r \in \mathcal {M} \end{array}} \frac{\chi (r)}{|r|} \sum _{a \hspace{0.05em}(\textrm{mod}\,g)} e \Big ( \frac{acr}{g} \Big )\\&= |g| \sum _{\begin{array}{c} r \hspace{0.05em}(\textrm{mod}\,g \ell ) \\ r \in \mathcal {M}\\ r \equiv 0 \hspace{0.05em}(\textrm{mod}\,g) \end{array}} \frac{\chi (r)}{|r|} = \chi (g) L(1,\chi ). \end{aligned}$$

Combining the last two equations above, the conclusion follows. $\square$

### Lemma 4.3

Under the same assumptions as Proposition 4.1 and the additional assumption that $\ell = 1$ (equivalently $\chi$ is principal), we have that

$$\begin{aligned} \mathscr {M}_{n} = \frac{q^n}{|g|} (n+1- 2\deg (g)), \end{aligned}$$

where $\mathscr {M}_n$ is as in (48).

### Proof

Recalling that $\mathcal {R}_2(u;\chi ,c/g)=\mathcal {D}_2(u;c/g)$ when $\ell =1$ and applying (33), we have that

$$\begin{aligned} \mathcal {D}_2 \Big ( u; \frac{c}{g}\Big )&= \sum _{a \hspace{0.05em}(\textrm{mod}\,g)} \mathcal {Z}(u;a,g) \mathcal {D}_1 \Big ( u ; \frac{ac}{g}\Big ) \\&= \sum _{a \hspace{0.05em}(\textrm{mod}\,g)} \Big [ \frac{u^{\deg (g)}}{1-qu}+ 1\!\!1_{a\in \mathcal {M}} u^{\deg (a)} \Big ] \mathcal {D}_1\Big ( u ; \frac{ac}{g}\Big ) \\&= \frac{u^{\deg (g)}}{(1-qu)^2} + \frac{u^{\deg (g)}}{1-qu}\sum _{\begin{array}{c} a \hspace{0.05em}(\textrm{mod}\,g) \\ a \ne 0 \end{array}} \mathcal {D}_1\Big ( u ; \frac{ac}{g}\Big ) + \sum _{\begin{array}{c} a \hspace{0.05em}(\textrm{mod}\,g) \\ a \in \mathcal {M} \end{array}} u^{\deg (a)} \mathcal {D}_1\Big ( u ; \frac{ac}{g}\Big )\\&= \frac{u^{\deg (g)}}{(1-qu)^2} + \frac{u^{\deg (g)}}{1-qu}\sum _{\begin{array}{c} a \hspace{0.05em}(\textrm{mod}\,g) \\ a \ne 0 \end{array}} \mathcal {D}_1\Big ( u ; \frac{a}{g}\Big ) + \sum _{\begin{array}{c} a \hspace{0.05em}(\textrm{mod}\,g) \\ a \in \mathcal {M} \end{array}} u^{\deg (a)} \mathcal {D}_1\Big ( u ; \frac{ac}{g}\Big ), \end{aligned}$$

where we used Lemma 3.1 and we made a change of variables in the last line. Using Lemma 3.1 again, it follows that $\mathcal {D}_1(u;ac/g)$ is a polynomial in *u*, so the third term in the expression above is a polynomial and there is no pole at $u=1/q$ in that case. It follows that

53 $$\begin{aligned} \mathscr {M}_n&=- {{\,\textrm{res}\,}}_{u = 1/q} \bigg ( \frac{u^{\deg (g)}}{(1-qu)^2} + \frac{u^{\deg (g)}}{1-qu}\sum _{\begin{array}{c} a \hspace{0.05em}(\textrm{mod}\,g) \\ a \ne 0 \end{array}} \mathcal {D}_1\Big ( u ; \frac{a}{g}\Big )\bigg ) \frac{1}{u^{n+1}} \nonumber \\&= ( n+1-\deg (g)) \frac{q^n}{|g|} + \frac{q^n}{|g|} \sum _{\begin{array}{c} a \hspace{0.05em}(\textrm{mod}\,g) \\ a \ne 0 \end{array}} \mathcal {D}_1\Big ( \frac{1}{q}; \frac{a}{g}\Big ). \end{aligned}$$

Now using Lemma 3.1 again,

$$\begin{aligned} \sum _{\begin{array}{c} a \hspace{0.05em}(\textrm{mod}\,g) \\ a \ne 0 \end{array}} \mathcal {D}_1\Big ( \frac{1}{q}; \frac{a}{g}\Big )&= \sum _{\begin{array}{c} a \hspace{0.05em}(\textrm{mod}\,g) \\ a \ne 0 \end{array}} \Big ( e_q ({{\,\textrm{sgn}\,}}(a)) + \deg (g) - \deg (a)-1\Big ) \\&= -\sum _{k=0}^{\deg (g)-1} q^k +(\deg (g)-1) (|g|-1) - (q-1) \sum _{k=0}^{\deg (g)-1} k q^k\\&= - \deg (g). \end{aligned}$$

Combining the above equation with (53), it follows that

$$\begin{aligned} \mathscr {M}_n = \frac{q^n}{|g|} (n+1-2\deg (g)). \end{aligned}$$

$$\square $$

We also have the following Proposition.

### Proposition 4.4

Let $g \in \mathcal {M}_{\geqslant 1}$, $\ell \in \mathcal {M}$ such that $\ell \not = 1$ and $\ell |g$, and $c \in \mathbb {F}_q[T]$ be such that $(c,g) = 1$ and let $r_\chi$ be as in (4) with $\chi$ a primitive Dirichlet character modulo $\ell$. Then,

$$\begin{aligned}  &   \sum _{f \in \mathcal {M}_n} r_\chi (f)e\Big (\frac{cf}{g}\Big ) = \frac{q^n}{|g|}G(\chi ) \overline{\chi }(c) L(1, \overline{\chi })\\    &   \quad \quad + \frac{q^{n}}{|g|} \overline{\chi }(-c) \sum _{\lambda \in \mathbb {F}_q^\times }\sum _{\eta _1,\eta _2\in \{0,1\}} \sum _{0 \leqslant k \leqslant \nu } b_{\nu -k}(\chi ) \sum _{f \in \mathcal {M}_{k}} r_{\chi }(f) e \Big ( \frac{ \lambda \overline{c} f}{g}\Big ), \end{aligned}$$

where $\nu =2\deg (g) -n-2$, and the coefficients $b_k(\chi )$ are defined in equation (49).

### Proof

We proceed as in the proof of Proposition 4.1, and we get that

54 $$\begin{aligned} \sum _{f \in \mathcal {M}_n} r_\chi (f)e\Big (\frac{cf}{g}\Big ) = \mathscr {M}_n + \mathscr {I}(-1,n), \end{aligned}$$

where, as before,

$$\begin{aligned} \mathscr {M}_n = - {{\,\textrm{res}\,}}_{u = 1/q} \bigg (\frac{\mathcal {R}_2(u;\chi ,c/g)}{u^{n+1}}\bigg ), \end{aligned}$$

and

$$\begin{aligned} \mathscr {I}(-1,n) = \frac{1}{2\pi i}\oint _{|u|=q} \mathcal {R}_2\Big (u;\chi ,\frac{c}{g}\Big ) \frac{du}{u^{n+1}}. \end{aligned}$$

Using Proposition 3.15 in the integrand above, we have that

$$\begin{aligned} \mathscr {I}(-1,n) = \frac{1}{2\pi i}\oint _{|u|=q} \overline{\chi }(-c) (qu^2)^{\deg (g) - 1} \sum _{\lambda \in \mathbb {F}_q^\times } \mathcal {B}_\lambda (u;\chi ) \mathcal {R}_2\Big (\frac{1}{qu};\chi ,\frac{\lambda \overline{c}}{g}\Big ) \frac{du}{u^{n+1}}. \end{aligned}$$

Making the change of variables $u=1/(qv)$, we get that

$$\begin{aligned} \mathscr {I}(-1,n) = q^{n+1-\deg (g)} \overline{\chi }(-c) \frac{1}{2 \pi i} \sum _{\lambda \in \mathbb {F}_q^\times }\oint _{|v|=q^{-2}} \mathcal {B}_\lambda \Big (\frac{1}{qv};\chi \Big ) \mathcal {R}_2\Big (v;\chi ,\frac{\lambda \overline{c}}{g}\Big ) \frac{dv}{v^{\nu +1}}, \end{aligned}$$

where $\nu =2\deg (g)-n-2$. Similarly as before, we have

$$\begin{aligned} \mathcal {B}_\lambda \Big (\frac{1}{qv};\chi \Big )=\frac{1}{q} \sum _{\eta _1,\eta _2 \in \{0,1\}} \sum _{k=0}^\infty b_k(\chi ) v^k, \end{aligned}$$

so

$$\begin{aligned} \mathscr {I}(-1,n) = q^{n+1-\deg (g)} \overline{\chi }(-c) \sum _{\lambda \in \mathbb {F}_q^\times }\sum _{\eta _1,\eta _2\in \{0,1\}}\sum _{k=0}^\infty \frac{b_k(\chi )}{2\pi i}\oint _{|v|=q^{-2}} \mathcal {R}_2\Big (v;\chi ,\frac{\lambda \overline{c}}{g}\Big ) \frac{dv}{v^{\nu - k + 1}}. \end{aligned}$$

Note that the integral above vanishes if $k \geqslant \nu +1$, and using Perron’s formula (14) it follows that

55 $$\begin{aligned} \mathscr {I}(-1,n) = q^{n+1-\deg (g)} \overline{\chi }(-c) \sum _{\lambda \in \mathbb {F}_q^\times }\sum _{\eta _1,\eta _2\in \{0,1\}} \sum _{0 \leqslant k \leqslant \nu } b_{\nu -k}(\chi ) \sum _{f \in \mathcal {M}_{k}} r_{\chi }(f) e \Big ( \frac{ \lambda \overline{c} f}{g}\Big ). \end{aligned}$$

Now we focus on the evaluation of $\mathscr {M}_n$. We rewrite

$$\begin{aligned} \mathcal {R}_2 \Big ( u ;\chi , \frac{c}{g} \Big )&= \sum _{a , b \hspace{0.05em}(\textrm{mod}\,g)} \sum _{\begin{array}{c} f_1 \equiv a \hspace{0.05em}(\textrm{mod}\,g) \\ f_2 \equiv b \hspace{0.05em}(\textrm{mod}\,g) \end{array}} \chi (f_1) e \Big ( \frac{abc}{g}\Big ) u^{\deg (f_1)+\deg (f_2)} \\&= \sum _{a,b \hspace{0.05em}(\textrm{mod}\,g)} \chi (a) e \Big ( \frac{abc}{g} \Big ) \Big [ \frac{u^{\deg (g)}}{1-qu}+ 1\!\!1_{a \in \mathcal {M}} u^{\deg (a)} \Big ]\Big [ \frac{u^{\deg (g)}}{1-qu} + 1\!\!1_{b \in \mathcal {M}} u^{\deg (b)} \Big ], \end{aligned}$$

where we used the fact that $\ell |g$ and Lemma 3.1. Computing the residue at $u=1/q$, it follows that

56 $$\begin{aligned} \mathscr {M}_n&= -\frac{q^n(2\deg (g)-n-1) }{|g|^2} \sum _{a , b \hspace{0.05em}(\textrm{mod}\,g)} \chi (a) e\Big ( \frac{abc}{g}\Big )+\frac{q^n}{|g|}\sum _{\begin{array}{c} a,b \hspace{0.05em}(\textrm{mod}\,g) \\ a \in \mathcal {M} \end{array}} \frac{\chi (a)}{|a|} e \Big (\frac{abc}{g} \Big ) \nonumber \\&+\frac{q^n}{|g|}\sum _{\begin{array}{c} a,b \hspace{0.05em}(\textrm{mod}\,g)\\ b \in \mathcal {M} \end{array}} \frac{\chi (a)}{|b|} e \Big (\frac{abc}{g} \Big ) . \end{aligned}$$

Now note that

$$\begin{aligned} \sum _{a,b \hspace{0.05em}(\textrm{mod}\,g)} \chi (a) e \Big ( \frac{abc}{g}\Big ) = \sum _{a \hspace{0.05em}(\textrm{mod}\,g)} \chi (a) |g| 1\!\!1_{a \equiv 0 \hspace{0.05em}(\textrm{mod}\,g)} = 0, \end{aligned}$$

where we again used the fact that $\ell |g$.

We similarly get that the second term in (56) vanishes. For the third term above, writing $a \equiv \alpha \hspace{0.05em}(\textrm{mod}\,\ell )$ and then $b=b_1 (g/\ell )$, we have

$$\begin{aligned} \sum _{\begin{array}{c} a , b \hspace{0.05em}(\textrm{mod}\,g) \\ b \in \mathcal {M} \end{array}}&\frac{\chi (a)}{|b|} e \Big ( \frac{abc}{g}\Big ) = \sum _{\alpha \hspace{0.05em}(\textrm{mod}\,\ell )} \chi (\alpha ) \sum _{\begin{array}{c} b \hspace{0.05em}(\textrm{mod}\,g) \\ b \in \mathcal {M} \end{array}} \frac{1}{|b|} e \Big ( \frac{bc \alpha }{g} \Big )\sum _{a_1 \hspace{0.05em}(\textrm{mod}\,g/\ell )} e \Big ( \frac{a_1 b c}{g/\ell } \Big ) \\&=\Big | \frac{g}{\ell } \Big | \sum _{\alpha \hspace{0.05em}(\textrm{mod}\,\ell )} \chi (\alpha ) \sum _{\begin{array}{c} b \hspace{0.05em}(\textrm{mod}\,g) \\ b \in \mathcal {M} \\ b \equiv 0 \hspace{0.05em}(\textrm{mod}\,g/\ell ) \end{array}} \frac{1}{|b|} e \Big ( \frac{bc \alpha }{g}\Big ) \\&= \sum _{\alpha \hspace{0.05em}(\textrm{mod}\,\ell )} \chi (\alpha ) \sum _{\begin{array}{c} b_1 \hspace{0.05em}(\textrm{mod}\,\ell ) \\ b_1 \in \mathcal {M} \end{array}} \frac{1}{|b_1|} e \Big ( \frac{b_1 c \alpha }{\ell }\Big ) = \sum _{\begin{array}{c} b_1 \hspace{0.05em}(\textrm{mod}\,\ell ) \\ b_1 \in \mathcal {M} \end{array}} \frac{1}{|b_1|} \sum _{\alpha \hspace{0.05em}(\textrm{mod}\,\ell )} \chi (\alpha ) e \Big ( \frac{b_1 c \alpha }{\ell }\Big )\\&= G(\chi ) \overline{\chi }(c) \sum _{\begin{array}{c} b_1 \hspace{0.05em}(\textrm{mod}\,\ell ) \\ b_1 \in \mathcal {M} \end{array}} \frac{\overline{\chi }(b_1)}{|b_1|} = G(\chi ) \overline{\chi }(c) L(1, \overline{\chi }). \end{aligned}$$

Using the two equations above in (56), we get that

$$\begin{aligned} \mathscr {M}_n = \frac{q^n}{|g|}G(\chi ) \overline{\chi }(c) L(1, \overline{\chi }). \end{aligned}$$

Using the above and equations (54) and (55), the conclusion follows. $\square$

## Setting up the proof when $\ell _2 \ne 1$

In this section, we assume that $\ell _2 \ne 1$ (so $\chi _2$ is a non-principal character modulo $\ell _2$). Let $d = \max \{m,n\}$, where $m=\deg (h)$. Observe that

$$\begin{aligned} r_{\chi _2}(f+h) = \sum _{\begin{array}{c} g \in \mathcal {M}_{\leqslant [d/2]}\\ g \mid (f+h) \end{array}} \chi _2(g) + \sum _{\begin{array}{c} g \in \mathbb {F}_q[T]\\ \deg (g) \leqslant [(d-1)/2]\\ \frac{f+h}{g}\in \mathcal {M} \end{array}} \chi _2\Big (\frac{f+h}{g}\Big ). \end{aligned}$$

Note that the second sum is not necessarily over *g* monic. In fact, if $m> n$, then ${{\,\textrm{sgn}\,}}(g)={{\,\textrm{sgn}\,}}(h)$ and if $m=n$, then ${{\,\textrm{sgn}\,}}(g)={{\,\textrm{sgn}\,}}(h)+1$.

For simplicity we assume first that $f+h$ is monic, that is, in the case when $m>n$, we assume that *h* is monic, and we further assume that $m\not =n$ (in the course of the proof, we will only briefly indicate after the appropriate formulas the changes that need to be made to deal with the case when $f+h$ is not monic). With this assumption, we have

$$\begin{aligned} r_{\chi _2}(f+h) = \sum _{\begin{array}{c} g \in \mathcal {M}_{\leqslant [d/2]}\\ g \mid (f+h) \end{array}} \chi _2(g) + \sum _{\begin{array}{c} g \in \mathcal {M}_{\leqslant [(d-1)/2]}\\ g \mid (f+h) \end{array}} \chi _2\Big (\frac{f+h}{g}\Big ). \end{aligned}$$

Using the above in the computation of the correlation, we find that

$$\begin{aligned} \sum _{f \in \mathcal {M}_n} r_{\chi _1}(f) r_{\chi _2}(f+h) = S_1 (\chi _1,\chi _2) + S_2 (\chi _1,\chi _2), \end{aligned}$$

where

$$\begin{aligned} S_1(\chi _1,\chi _2) = \sum _{f \in \mathcal {M}_n} r_{\chi _1}(f)\sum _{\begin{array}{c} g \in \mathcal {M}_{\leqslant [d/2]}\\ g \mid (f+h) \end{array}} \chi _2(g), \end{aligned}$$

and

$$\begin{aligned} S_2(\chi _1,\chi _2) =\sum _{f \in \mathcal {M}_n} r_{\chi _1}(f) \sum _{\begin{array}{c} g \in \mathcal {M}_{\leqslant [(d-1)/2]}\\ g \mid (f+h) \end{array}} \chi _2\Big (\frac{f+h}{g}\Big ). \end{aligned}$$

From now on, for ease of notation, we will denote $S_i(\chi _1,\chi _2)$ by $S_i$ for $i=1,2$.

We rewrite

$$\begin{aligned} S_1 = \sum _{g \in \mathcal {M}_{\leqslant [d/2]}} \chi _2(g) \sum _{\begin{array}{c} f \in \mathcal {M}_n\\ f \equiv -h \hspace{0.05em}(\textrm{mod}\,g) \end{array}} r_{\chi _1}(f). \end{aligned}$$

Detecting the congruence condition using additive characters modulo *g*, we get that

$$\begin{aligned} S_1 =\sum _{g \in \mathcal {M}_{\leqslant [d/2]}} \frac{\chi _2(g)}{|g|} \sum _{b \hspace{0.05em}(\textrm{mod}\,g)} e \Big ( \frac{bh}{g} \Big ) \sum _{f \in \mathcal {M}_n} r_{\chi _1}(f) e \Big ( \frac{bf}{g}\Big ). \end{aligned}$$

We further rewrite $S_1$ as57For $S_2$, we interchange the *f* and *g* sums as before, and arrange the terms according to the congruence class modulo $\ell _2$ that $(f+h)/g$ takes. In particular, $(f+h)/g \equiv a \hspace{0.05em}(\textrm{mod}\,\ell _2)$ implies that $f \equiv -h + ag \hspace{0.05em}(\textrm{mod}\,g\ell _2)$. This latter congruence entails the fact that $g \mid (f+h)$, so we omit the constraint. In doing so, we find that

$$\begin{aligned} S_2 = \sum _{a \hspace{0.05em}(\textrm{mod}\,\ell _2)} \chi _2(a) \sum _{g \in \mathcal {M}_{\leqslant [(d-1)/2]}} \sum _{\begin{array}{c} f \in \mathcal {M}_n \\ f \equiv -h + ag \hspace{0.05em}(\textrm{mod}\,g\ell _2) \end{array}} r_{\chi _1}(f). \end{aligned}$$

Detecting the congruence using additive characters modulo $g\ell _2$, we find that

$$\begin{aligned} S_2 = \frac{1}{|\ell _2|} \sum _{a \hspace{0.05em}(\textrm{mod}\,\ell _2)} \chi _2(a) \sum _{g \in \mathcal {M}_{\leqslant [(d-1)/2]}} \frac{1}{|g|} \sum _{b \hspace{0.05em}(\textrm{mod}\,g\ell _2)} e\Big (\frac{bh -abg}{g\ell _2}\Big ) \sum _{f \in \mathcal {M}_n} r_{\chi _1}(f) e\Big (\frac{bf}{g\ell _2}\Big ). \end{aligned}$$

Focusing only on the sum over *a*, we observe that we have

$$\sum _{a\hspace{0.05em}(\textrm{mod}\,\ell _2)} \chi _2(a) e\Big (\frac{-ab}{\ell _2} \Big ) = \overline{\chi _2}(-b) G(\chi _2). $$

Note that this identity is valid even when $\ell _2 \mid b$.

Thus, we have obtained,

$$\begin{aligned} S_2 = \frac{G(\chi _2)}{|\ell _2|} \sum _{g \in \mathcal {M}_{\leqslant [(d-1)/2]}} \frac{1}{|g|} \sum _{b \hspace{0.05em}(\textrm{mod}\,g\ell _2)} \overline{\chi _2}(-b) e\Big (\frac{bh}{g\ell _2}\Big ) \sum _{f \in \mathcal {M}_n} r_{\chi _1}(f) e\Big (\frac{bf}{g\ell _2}\Big ). \end{aligned}$$

To apply the functional equation of $\mathcal {R}_2$ to the innermost sum, we need to reduce $\frac{b}{g\ell _2}$. To do this, first observe that $\chi _2(-b)$ ensures $\ell _2 \not \mid b$. Thus, we must have

$$\begin{aligned} \frac{b}{g\ell _2} = \frac{b_1}{g_1\ell _2}, \end{aligned}$$

where $\ell _2 \not \mid \frac{g}{g_1}$ (and the latter fraction is reduced). Further, for every reduced fraction of this type, there is exactly one choice of *b* for which it occurs. Thus, we may rewrite the sum over $b\hspace{0.05em}(\textrm{mod}\,g\ell _2)$ as a sum over $g_1 \mid g$ satisfying $\ell _2 \not \mid \frac{g}{g_1}$ and a sum over reduced residues $b_1 \hspace{0.05em}(\textrm{mod}\,g_1\ell _2)$.

Upon making these substitutions, we find thatBy multiplicativity, we have $\chi _2(-b_1g/g_1) = \chi _2(-b_1) \chi _2(g/g_1)$. In particular, we see that we can drop the constraint $\ell _2 \not \mid \frac{g}{g_1}$ from the sum over $g_1$. This gives58

### The case $\ell _1 \ne 1$

Here, we assume that $\ell _1 \ne 1$. We first rewrite $S_1$ as follows. In equation (57), we apply the Voronoi summation formulas as in Propositions 4.1 and 4.4. In this case, we write

$$\begin{aligned} S_1 = S_{11}+S_{12}, \end{aligned}$$

where $S_{11}$ corresponds to the sum over $\ell _1 \not \mid g_1$ and $S_{12}$ corresponds to the sum over $\ell _1 | g_1$. Applying the Voronoi summation formula from Proposition 4.1 and Lemma 4.2, we write

$$\begin{aligned} S_{11}&= M_{11}+E_{11}, \end{aligned}$$

where59and60where $$\mu = 2\deg (g_1) + \deg (\ell _1)-n-2$$.

#### Remark 5.1

The error term $E_{11}$ vanishes in the following cases:

- when $m \leqslant n$, *n* is even, and $\deg (\ell _1)=1$;
- when $m \leqslant n$, *n* is odd, and $\deg (\ell _1)<3$;
- when $m=n+1$, *n* is even, and $\deg (\ell _1)=1$;

where we recall that $\deg (\ell _1)> 0$ since $\ell _1\not = 1$.

Applying the Voronoi summation formula from Proposition 4.4, we have

$$\begin{aligned} S_{12}=M_{12}+E_{12}, \end{aligned}$$

where61and62where $$\nu =2\deg (g_1)-n-2$$.

#### Remark 5.2

The error term $E_{12}$ vanishes when $m<n+2$ or when $m=n+2$ and *n* is odd.

#### Remark 5.3

If $\ell _1=\ell _2=\ell$, then $\ell \mid g_1$ implies that $\ell \mid g$ and we get $M_{12}=E_{12}=0$.

Now we focus on $S_2$ given by equation (58). We will use the Voronoi summation formula for the sum over *f*. We first assume that $\ell _1 \ne \ell _2$. We write

$$\begin{aligned} S_2 = S_{21}+S_{22}, \end{aligned}$$

where $S_{21}$ corresponds to the case $\ell _1 \not \mid g_1$ and $S_{22}$ corresponds to the case $\ell _1|g_1$. Using Proposition 4.1 and Lemma 4.2, we write

$$\begin{aligned} S_{21}=M_{21}+E_{21}, \end{aligned}$$

where63and64where $$\mu = 2 \deg (g_1 \ell _2)+\deg (\ell _1)-n-2$$.

#### Remark 5.4

The error term $E_{21}$ vanishes when $m\leqslant n$, *n* is even, and $\deg (\ell _1)=\deg (\ell _2)=1$. We recall that $\deg (\ell _1), \deg (\ell _2)>0$ since $\ell _1, \ell _2\not = 1$.

Now using Proposition 4.4, we further rewrite65and66where $\nu = 2 \deg (g_1 \ell _2)-n-2$.

#### Remark 5.5

The error term $E_{22}$ vanishes when $m\leqslant n$ and $\deg (\ell _2)=1$, or when $m=n+1$, *n* is odd, and $\deg (\ell _2)=1$. (Recall that here $\deg (\ell _2)>0.$)

Now we assume that $\ell _1 = \ell _2:=\ell$. Since $\ell \mid (g_1\ell )$ trivially, we use Proposition 4.4. In this case, we write

$$\begin{aligned} S_2 = M_2+E_2, \end{aligned}$$

and $M_2$ is now similar to $M_{22}$ and $E_2$ is similar to $E_{22}$. Namely, we have67and68where $\nu = 2 \deg (g_1 \ell )-n-2$.

#### Remark 5.6

As before, the error term $E_2$ vanishes when $m \leqslant n$ and $\deg (\ell )<2$ or when $m=n+1$, *n* s odd, and $\deg (\ell )<2$. (Recall that here $\deg (\ell )>0$.)

### The case $\ell _1 =1$

If $\ell _1=1$, using equation (57), we haveUsing Proposition 4.1 and Lemma 4.3, we write $S_1= M_1+E_1$, where69and70with $\mu =2 \deg (g_1)-n-2$.

#### Remark 5.7

As before, the error term $E_1$ vanishes when $m < n+2$ or when $m=n+2$ and *n* is odd.

In the case of $S_2$, given in equation (58), we use Proposition 4.1 and Lemma 4.3 again, and we write $S_2=M_2+E_2,$ where71and72with $\mu = 2\deg (g_1 \ell _2) - n - 2$.

#### Remark 5.8

As before, the error term $E_2$ vanishes when $m \leqslant n$ and $\deg (\ell _2)<2$ or when $m=n+1$, *n* s odd, and $\deg (\ell _2)<2$. (Recall that here $\deg (\ell _2)>0$.)

## Evaluating the main terms when $\ell _2 \ne 1$

### The main terms when $\ell _1 \ne 1$

Here, we will evaluate the main terms $M_{11}$, $M_{12}$, $M_{21}$, and $M_{22}$ in the case where $\ell _1 \ne 1$.

We begin by evaluating $M_{11}$, given in (59). Recall that expression (59) holds regardless whether $\ell _1 = \ell _2$ or not. Note that we have73and hence, writing $g_1=Bg_2$ and $g=Ag_1$,

74 $$\begin{aligned} M_{11}&= q^n L(1,\chi _1) \sum _{g \in \mathcal {M}_{\leqslant [d/2]}} \frac{\chi _2(g)}{|g|} \sum _{\begin{array}{c} g_1|g\\ \ell _1\not \mid g_1 \end{array}} \frac{\chi _1(g_1)}{|g_1|} \sum _{g_2|(g_1,h)} \mu \Big (\frac{g_1}{g_2}\Big ) |g_2| \nonumber \\&= q^n L(1,\chi _1) \sum _{g_2|h} \frac{(\chi _1 \chi _2)(g_2)}{|g_2|} \sum _{\deg (AB) \leqslant [d/2]-\deg (g_2)} \frac{\mu (B)\chi _2(A) (\chi _1 \chi _2)(B) }{|AB^2|}. \end{aligned}$$

Using Perron’s formula (14) twice for the sums over *A* and *B*, we get that

$$\begin{aligned} M_{11} = q^n L(1,\chi _1) \frac{1}{(2 \pi i)^2} \oint _{|y|=q^{-\epsilon }} \oint _{|x|=q^{-\epsilon }} \frac{ \mathcal {L} \left( {xy}/{q},\chi _2\right) }{\mathcal {L} \left( {x}/{q^2},\chi _1 \chi _2\right) (1-x)(1-y)(xy)^{[d/2]}} \\ \times \sum _{g_2|h} \frac{(\chi _1 \chi _2)(g_2) (xy)^{\deg (g_2)}}{|g_2|} \, \frac{dx}{x} \frac{dy}{y}. \end{aligned}$$

In the integral over *x*, we shift the contour of integration to $|x|=q^{1/2}$ and encounter a pole at $x=1$. The integral over the new contour is bounded up to a constant by $q^{n-d/4+\epsilon d}$. We then get that

$$\begin{aligned} M_{11}&= \frac{ q^n L(1,\chi _1)}{L(2,\chi _1\chi _2)} \frac{1}{2 \pi i} \oint _{|y|=q^{-\epsilon }} \frac{ \mathcal {L} \left( {y}/{q}, \chi _2\right) }{ (1-y)y^{[d/2]}} \sum _{g_2|h} \frac{(\chi _1 \chi _2)(g_2) y^{\deg (g_2)}}{|g_2|} \frac{dy}{y} + O\Big (q^{n-d/4+\epsilon d} \Big ). \end{aligned}$$

Now in the integral over *y* we shift the contour of integration to $|y|=q^{1/2}$ and encounter the pole at $y=1$. By the Lindelöf hypothesis (13), the integrand on the circle integral over the new contour is bounded up to a constant by $q^{n-d/4+\epsilon d}$. It follows that

75 $$\begin{aligned} M_{11} =\frac{ q^n L(1,\chi _1)L(1,\chi _2)}{L(2,\chi _1\chi _2)} \sum _{\begin{array}{c} g_2|h \\ \deg (g_2) \leqslant [d/2] \end{array}} \frac{ (\chi _1 \chi _2)(g_2)}{|g_2|}+O\Big (q^{n-d/4+\epsilon d} \Big ). \end{aligned}$$

We now focus on evaluating $M_{12}$, which is given by equation (61). As remarked before, if $\ell _1=\ell _2$, then $M_{12}=0$. We then assume that $\ell _1 \ne \ell _2$. We rewriteWe have76In the sum above, note that we need $\ell _1 |g_3$, since otherwise $\overline{\chi _1}(\ell _1g_2/g_3)=0$; hence we write $g_3=\ell _1 g_4$. Writing $b_1=\alpha +b_2\ell _1$, we then get that

$$\begin{aligned} (76)&= \sum _{g_4|g_2} \mu \Big (\frac{g_2}{g_4}\Big ) \overline{\chi _1}\Big (\frac{g_2}{g_4}\Big ) \sum _{b_1 \hspace{0.05em}(\textrm{mod}\,\ell _1 g_4)} \overline{\chi _1}(b_1) e \Big ( \frac{b_1 h}{\ell _1 g_4}\Big ) \\&= \sum _{g_4|g_2} \mu \Big (\frac{g_2}{g_4}\Big ) \overline{\chi _1}\Big (\frac{g_2}{g_4}\Big ) \sum _{\alpha \hspace{0.05em}(\textrm{mod}\,\ell _1)} \overline{\chi _1}(\alpha ) e \Big ( \frac{\alpha h}{\ell _1 g_4} \Big ) \sum _{b_2 \hspace{0.05em}(\textrm{mod}\,g_4)} e \Big ( \frac{b_2 h }{g_4} \Big ) \\&= \sum _{g_4|(g_2,h)} |g_4|\mu \Big (\frac{g_2}{g_4}\Big ) \overline{\chi _1}\Big (\frac{g_2}{g_4}\Big ) \sum _{\alpha \hspace{0.05em}(\textrm{mod}\,\ell _1)} \overline{\chi _1}(\alpha ) e \Big ( \frac{\alpha h/g_4}{\ell _1 } \Big ) \\&= G(\overline{\chi _1}) \sum _{g_4|(g_2,h)} |g_4| \mu \Big (\frac{g_2}{g_4}\Big ) \overline{\chi _1}\Big (\frac{g_2}{g_4}\Big ) \chi _1\Big (\frac{h}{g_4}\Big ). \end{aligned}$$

Now, using the above and the fact that $G(\chi _1) G(\overline{\chi _1})= |\ell _1|$, and writing $g_2=Bg_4$ and $\frac{g}{\ell _1}=Cg_4$ so that $B\mid C$, we get that

$$\begin{aligned}&M_{12} = q^n L(1,\overline{\chi _1}) \sum _{\begin{array}{c} g \in \mathcal {M}_{\leqslant [d/2]} \\ \ell _1 | g \end{array}} \frac{\chi _2(g)}{|g|} \sum _{g_2| (g/\ell _1)} \frac{1}{|g_2|}\sum _{g_4|(g_2,h)} |g_4|\mu \Big (\frac{g_2}{g_4}\Big ) \overline{\chi _1}\Big (\frac{g_2}{g_4}\Big ) \chi _1\Big (\frac{h}{g_4}\Big )\\&= \frac{q^n \chi _2(\ell _1)L(1,\overline{\chi _1}) }{|\ell _1|} \sum _{g_4|h} \frac{ \chi _2(g_4) \chi _1(h/g_4)}{|g_4|} \sum _{\begin{array}{c} C \in \mathcal {M}\\ \deg (C) \leqslant [d/2]-\deg (\ell _1)-\deg (g_4) \end{array}} \frac{\chi _2(C)}{|C|} \sum _{B|C} \frac{\mu (B)\overline{\chi _1}(B) }{|B|} \\&= \frac{q^n\chi _2(\ell _1)L(1,\overline{\chi _1}) }{|\ell _1|} \sum _{g_4|h} \frac{\chi _2(g_4) \chi _1(h/g_4)}{|g_4|} \sum _{\begin{array}{c} C \in \mathcal {M}\\ \deg (C) \leqslant [d/2]-\deg (\ell _1)-\deg (g_4) \end{array}} \frac{\chi _2(C)}{|C|} \prod _{P|C} \Big ( 1 - \frac{\overline{\chi _1}(P) }{|P|}\Big ). \end{aligned}$$

Looking at the generating series for the sum over *C*, we have

$$\begin{aligned} \sum _{C \in \mathcal {M}} \frac{\chi _2(C)}{|C|} \prod _{P|C} \Big ( 1 - \frac{\overline{\chi _1}(P) }{|P|}\Big ) u^{\deg (C)} = \frac{\mathcal {L}(u/q,\chi _2)}{\mathcal {L}(u/q^2,\overline{\chi _1} \chi _2)}. \end{aligned}$$

Using Perron’s formula (14), it follows that

$$\begin{aligned}  &   \sum _{C \in \mathcal {M}_{\leqslant [d/2]-\deg (\ell _1)-\deg (g_4)}} \frac{\chi _2(C)}{|C|} \prod _{P|C} \Big ( 1 - \frac{\overline{\chi _1}(P) }{|P|}\Big )\\  &   \quad \quad = \frac{1}{2 \pi i} \oint _{|u|=q^{-\epsilon }} \frac{\mathcal {L}(u/q,\chi _2)}{\mathcal {L}(u/q^2,\overline{\chi _1} \chi _2)(1-u)u^{[d/2]-\deg (\ell _1) - \deg (g_4)}} \frac{du}{u}. \end{aligned}$$

In the integral above, we shift the contour of integration to $|u|=q^{1/2}$ and encounter a pole at $u=1$. Using the Lindelöf hypothesis (13) to bound the integrand on the circle $|u|=q^{1/2}$ and evaluating the residue at $u=1$, it follows that

$$\begin{aligned} \sum _{C \in \mathcal {M}_{\leqslant [d/2]-\deg (\ell _1)-\deg (g_4)}}&\frac{\chi _2(C)}{|C|} \prod _{P|C} \Big ( 1 - \frac{\overline{\chi _1}(P) }{|P|}\Big ) = \frac{\mathcal {L}(1/q,\chi _2)}{\mathcal {L}(1/q^2,\overline{\chi _1} \chi _2)} + O \Big ( \frac{|\ell _2|^{\epsilon } \sqrt{|\ell _1 g_4|}}{q^{d/4}}\Big ) . \end{aligned}$$

Using the above in the expression for $M_{12}$, we get that

77 $$\begin{aligned} M_{12} = \frac{q^n \chi _2(\ell _1) L(1,\overline{\chi _1}) L(1,\chi _2)}{|\ell _1|L(2,\overline{\chi _1} \chi _2)} \sum _{\begin{array}{c} g_4|h \\ \deg (g_4) \leqslant [d/2]-\deg (\ell _1) \end{array}} \frac{\chi _2(g_4) \chi _1(h/g_4)}{|g_4|} + O \Big ( \frac{q^{n-d/4} |h \ell _2|^{\epsilon }}{|\ell _1|^{1/2}} \Big ). \end{aligned}$$

Now we will evaluate the terms $M_{21}$ and $M_{22}$ in the case $\ell _1 \ne \ell _2$. Using the computation in (76), we have thatso using equation (63) and the fact that $G(\chi _2) G(\overline{\chi _2})=|\ell _2|$, we get that

78 $$\begin{aligned} M_{21} = \frac{q^n \chi _1(\ell _2) \overline{\chi _2}(-1) L(1,\chi _1) }{|\ell _2|} \sum _{g \in \mathcal {M}_{\leqslant [(d-1)/2]}} \frac{1}{|g|} \sum _{g_1 \mid g} \frac{\overline{\chi _2}({g}/{g_1}) \chi _1(g_1)}{|g_1|} \nonumber \\ \times \sum _{g_3|(g_1,h)} |g_3| \mu \Big (\frac{g_1}{g_3}\Big ) \overline{\chi _2}\Big (\frac{g_1}{g_3}\Big ) \chi _2\Big (\frac{h}{g_3}\Big ). \end{aligned}$$

When $f+h$ is not monic, $M_{21}$ in (78) does not change for $m>n$ and is multiplied by $\chi _2({{\,\textrm{sgn}\,}}(h))\overline{\chi _2}({{\,\textrm{sgn}\,}}(h)+1)$ for $m=n$.

Setting $g_1=g_3 A$ and $g=g_1 B$, we have

$$\begin{aligned} M_{21}= &   \frac{q^n \chi _1(\ell _2) \overline{\chi _2}(-1) L(1,\chi _1)}{|\ell _2|} \sum _{g_3|h} \frac{\chi _1(g_3) \chi _2(h/g_3)}{|g_3|}\\  &   \quad \quad \quad \times \sum _{\deg (AB) \leqslant [(d-1)/2]-\deg (g_3)} \frac{\mu (A) \chi _1 \overline{\chi _2}(A) \overline{\chi _2}(B)}{|A^2B|}. \end{aligned}$$

Applying Perron’s formula twice for the sums over *A*, *B*, we get that

79 $$\begin{aligned}&\sum _{\deg (AB) \leqslant [(d-1)/2]-\deg (g_3)} \frac{\mu (A) \chi _1 \overline{\chi _2}(A) \overline{\chi _2}(B)}{|A^2B|} \nonumber \\&= \frac{1}{(2 \pi i)^2}\oint _{|y|=q^{-\epsilon }} \oint _{|x|=q^{-\epsilon }} \frac{ \mathcal {L}(x/q,\overline{\chi _2})}{\mathcal {L}(xy/q^2,\chi _1 \overline{\chi _2})(1-x)(1-y)(xy)^{[(d-1)/2]-\deg (g_3)}} \frac{dx}{x} \frac{dy}{y}. \end{aligned}$$

In the integral over *x*, we shift the contour of integration to $|x|=q^{1/2}$, and encounter the pole at $x=1$. By applying the Lindelöf hypothesis (13), the error term coming from the double integral over the new contour is bounded by $q^{n-d/4-\deg (\ell _2)+\epsilon d}$. We further shift the contour over *y* to $|y|=q^{1/2}$, and the error term coming from the new single integral will be similarly bounded by $q^{n-d/4-\deg (\ell _2)+\epsilon d}$. Overall, it follows that

80 $$\begin{aligned} M_{21} = \frac{q^n \chi _1(\ell _2) \overline{\chi _2}(-1) L(1,\chi _1) L(1,\overline{\chi _2})}{|\ell _2|L(2,\chi _1 \overline{\chi _2})} \sum _{\begin{array}{c} g_3|h \\ \deg (g_3) \leqslant [(d-1)/2] \end{array}} \frac{\chi _1(g_3) \chi _2(h/g_3)}{|g_3|}\nonumber \\ + O \Big (q^{n-d/4-\deg (\ell _2)+\epsilon d} \Big ). \end{aligned}$$

Now we evaluate $M_{22}$ given in (65).Using the above in the expression (65) for $M_{22}$ and setting $g_1=\ell _1 g_2$, we have

81 $$\begin{aligned} M_{22}&= \frac{q^n \overline{\chi _2}(-1)G(\chi _1) G(\chi _2) G(\overline{\chi _1 \chi _2})L(1,\overline{\chi }_1) }{|\ell _2|^2} \sum _{g \in \mathcal {M}_{\leqslant [(d-1)/2]}} \frac{1}{|g|} \sum _{\begin{array}{c} g_1 \mid g \\ \ell _1|g_1 \end{array}} \frac{\overline{\chi _2}({g}/{g_1})}{|g_1|} \nonumber \\&\times \sum _{\begin{array}{c} g_5| (g_1/\ell _1) \\ g_5|h \end{array}} |g_5| \mu \Big (\frac{g_1/\ell _1}{g_5}\Big )\overline{\chi _1 \chi _2}\Big (\frac{g_1/\ell _1}{g_5}\Big ) \chi _1 \chi _2\Big (\frac{h}{g_5}\Big ) \nonumber \\  &= \frac{q^n \overline{\chi _2}(-1) G(\chi _1) G(\chi _2) G(\overline{\chi _1 \chi _2})L(1,\overline{\chi }_1)}{|\ell _1 \ell _2^2|} \nonumber \\&\times \sum _{\begin{array}{c} g \in \mathcal {M}_{\leqslant [(d-1)/2]}\\ \ell _1|g \end{array}} \frac{1}{|g|} \sum _{g_2|(g/\ell _1)} \frac{\overline{\chi _2}({g}/{ (\ell _1 g_2)})}{|g_2|} \sum _{\begin{array}{c} g_5| g_2 \\ g_5|h \end{array}} |g_5| \mu \Big (\frac{g_2}{g_5}\Big )\overline{\chi _1 \chi _2}\Big (\frac{g_2}{g_5}\Big ) \chi _1 \chi _2\Big (\frac{h}{g_5}\Big )\nonumber \\&= \frac{q^n \overline{\chi _2}(-1) G(\chi _1) G(\chi _2) G(\overline{\chi _1 \chi _2})L(1,\overline{\chi }_1)}{|\ell _1^2 \ell _2^2|} \sum _{g_5|h} \frac{\chi _1 \chi _2(h/g_5)}{|g_5|}\nonumber \\&\times \sum _{\deg (AB) \leqslant [(d-1)/2]-\deg (\ell _1 g_5)} \frac{\mu (A) \overline{\chi _1 \chi _2}(A) \overline{\chi _2}(B)}{|A^2B|}, \end{aligned}$$

where in the last equality, we set $g_2=g_5A$ and $g=\ell _1g_2B$.

When $f+h$ is not monic, $M_{22}$ in (81) is multiplied by $\chi _1({{\,\textrm{sgn}\,}}(h))$ for $m>n$ and by $\chi _1\chi _2({{\,\textrm{sgn}\,}}(h))\overline{\chi _2}\left( {{\,\textrm{sgn}\,}}(h)+1\right)$ for $m=n$.

We proceed as before, applying Perron’s formula as in (79) and using the fact that

$$\begin{aligned} G(\overline{\chi _1 \chi _2}) = \overline{\chi _1}(\ell _2) \overline{\chi _2}(\ell _1) G(\overline{\chi _1}) G(\overline{\chi _2}), \end{aligned}$$

which follows from the Chinese Remainder Theorem, to obtain that

82 $$\begin{aligned} M_{22}= \frac{q^n \overline{\chi _1}(\ell _2) \overline{\chi _2}(-\ell _1) L(1,\overline{\chi }_1)L(1,\overline{\chi _2})}{|\ell _1 \ell _2 | L(2,\overline{\chi _1 \chi _2})} \sum _{\begin{array}{c} g_5|h \\ \deg (g_5) \leqslant [(d-1)/2]-\deg (\ell _1) \end{array}} \frac{ \chi _1 \chi _2(h/g_5)}{|g_5|}\nonumber \\ +O \Big ( q^{n-d/4-\deg (\ell _1)/2-\deg (\ell _2)+\epsilon d} \Big ). \end{aligned}$$

Now we evaluate $M_2$, the main term coming from $S_2$ in the case that $\ell _1=\ell _2$. Proceeding with equation (67) as in equation (76) and applying Perron’s formula as in (79), we have

83 $$\begin{aligned} M_2&= \frac{q^n \overline{\chi _2}(-1) G(\chi _1) G(\chi _2)G(\overline{\chi _1 \chi _2})L(1,\overline{\chi }_1)}{|\ell |^2} \sum _{g \in \mathcal {M}_{\leqslant [(d-1)/2]}} \frac{1}{|g|} \sum _{\begin{array}{c} g_1 \mid g \end{array}} \frac{\overline{\chi _2}({g}/{g_1})}{|g_1|} \nonumber \\  &\times \sum _{g_4|(g_1,h)} |g_4| \mu \Big (\frac{g_1}{g_4}\Big ) \overline{\chi _1 \chi _2}\Big (\frac{g_1}{g_4}\Big ) \chi _1 \chi _2\Big (\frac{h}{g_4}\Big ) \end{aligned}$$

84 $$\begin{aligned}&= \frac{q^n \overline{\chi _2}(-1) G(\chi _1) G(\chi _2)G(\overline{\chi _1 \chi _2})L(1,\overline{\chi }_1)}{|\ell |^2} \sum _{g_4|h} \frac{\chi _1 \chi _2(h/g_4)}{|g_4|} \nonumber \\&\times \sum _{\deg (AB) \leqslant [(d-1)/2]-\deg (g_4)} \frac{\mu (A) \overline{\chi _1 \chi _2}(A) \overline{\chi _2}(B)}{|A^2B|} \nonumber \\&= \frac{q^n \overline{\chi _2}(-1) G(\chi _1) G(\chi _2)G(\overline{\chi _1 \chi _2})L(1,\overline{\chi }_1)L(1,\overline{\chi _2})}{|\ell ^2|L(2,\overline{\chi _1 \chi _2})} \sum _{\begin{array}{c} g_4| h \\ \deg (g_4) \leqslant [(d-1)/2] \end{array}} \frac{ \chi _1 \chi _2(h/g_4)}{|g_4|}\nonumber \\  &\quad \quad + O \Big (q^{n-d/4-\deg (\ell )/2+\epsilon d} \Big ), \end{aligned}$$

where we have set $g_1=g_2A$ and $g=g_1B$. When $f+h$ is not monic, $M_{2}$ in (83) is multiplied by $\chi _1({{\,\textrm{sgn}\,}}(h))$ for $m>n$ and is multiplied by $\chi _1\chi _2({{\,\textrm{sgn}\,}}(h))\overline{\chi _2}\left( {{\,\textrm{sgn}\,}}(h)+1\right)$ for $m=n$.

### The main terms when $\ell _1=1$

To evaluate the main term $M_1$ given by (69), we proceed as before, using (73), and applying Perron’s formula as in (79), we have

85 $$\begin{aligned} M_1&=q^n \sum _{g \in \mathcal {M}_{\leqslant [d/2]}} \frac{\chi _2(g)}{|g|} \sum _{g_1|g} \frac{n+1-2\deg (g_1)}{|g_1|} \sum _{g_2|(g_1,h)} \mu \Big (\frac{g_1}{g_2}\Big ) |g_2| \nonumber \\&= q^n \sum _{g \in \mathcal {M}_{\leqslant [d/2]}} \frac{\chi _2(g)}{|g|} \frac{1}{2 \pi i} \oint _{|u|=q^{-\epsilon }} \sum _{g_1|g}\frac{du}{|g_1|(1-u)^2 u^{n+1-2\deg (g_1)}} \sum _{g_2|(g_1,h)} \mu \Big (\frac{g_1}{g_2}\Big ) |g_2| \nonumber \\&= \frac{q^n}{(2 \pi i)^3} \oint _{|y|=q^{-\epsilon }} \oint _{|x|=q^{-\epsilon }} \oint _{|u|=q^{-\epsilon }} \frac{ \mathcal {L}(x/q,\chi _2)}{\mathcal {L}( {xyu^2}/{q^2}, \chi _2 ) (1-u)^2 (1-x)(1-y)(xy)^{[d/2]}u^{n}} \nonumber \\  &\times \sum _{g_2|h} \frac{\chi _2(g_2)(xyu^2)^{\deg (g_2)}}{|g_2| } \frac{du}{u} \frac{dx}{x} \frac{dy}{y}. \end{aligned}$$

In the integral over *y* we shift the contour of integration to $|y|=q$ and we encounter a pole at $y=1$. The integral over the new contour will be bounded by $q^{n-d/2+\epsilon n}$. Then we have

$$\begin{aligned} M_1 = \frac{q^n}{(2 \pi i)^2} \oint _{|x|=q^{-\epsilon }} \oint _{|u|=q^{-\epsilon }} \frac{ \mathcal {L}(x/q,\chi _2)}{\mathcal {L}({xu^2}/{q^2}, \chi _2) (1-u)^2 (1-x)x^{[d/2]}u^{n}}\\ \times \sum _{g_2|h} \frac{\chi _2(g_2)(u^2 x)^{\deg (g_2)}}{|g_2| } \frac{du}{u} \frac{dx}{x} + O \Big ( q^{n-d/2+\epsilon n}\Big ). \end{aligned}$$

In the integral over *x* we shift the contour to $|x|=q$ and encounter the pole at $x=1$. The integral over the new contour will be bounded by $q^{n-d/2+\deg (\ell _2)/2+\epsilon n}$. Then we obtain

86 $$\begin{aligned} M_1&= q^n L(1,\chi _2) \frac{1}{2 \pi i} \oint _{|u|=q^{-\epsilon }} \frac{ 1}{\mathcal {L}( {u^2}/{q^2}, \chi _2 ) (1-u)^2 u^{n}} \sum _{g_2|h} \frac{\chi _2(g_2)u^{2\deg (g_2)}}{|g_2| } \frac{du}{u} \nonumber \\&\quad +O \Big (q^{n-d/2+\epsilon n} |\ell _2|^{1/2} \Big ) \nonumber \\&=\frac{q^n L(1,\chi _2)}{L(2,\chi _2)} \sum _{\begin{array}{c} g_2|h \\ \deg (g_2) \leqslant [n/2] \end{array}} \frac{\chi _2(g_2)}{|g_2|} \Big [ n+1-2\deg (g_2)- \frac{2L'(2,\chi _2)}{(\log q) L(2,\chi _2)}\Big ]\nonumber \\&\quad + O \Big (q^{n-d/2+\deg (\ell _2)/2+\epsilon n} +q^{n/2+\epsilon n} \Big ), \end{aligned}$$

where in the final step we shifted the last integral to $|u|=q^{1/2}$ resulting in a contribution from the residue at $u=1$ and an error term bounded by $q^{n/2+\epsilon n}$.

Now we will evaluate the term $M_2$ given by (71). We proceed as before (see (76)). We have

87 $$\begin{aligned} M_2&=\frac{q^n\overline{\chi _2}(-1)}{|\ell _2|} \sum _{g \in \mathcal {M}_{\leqslant [(d-1)/2]}} \frac{1}{|g|} \sum _{g_1 \mid g} \frac{\overline{\chi _2}({g}/{g_1})(n+1-2\deg (g_1 \ell _2))}{|g_1|} \nonumber \\  &\quad \times \sum _{g_4|(g_1,h)} |g_4| \mu \Big (\frac{g_1}{g_4}\Big ) \overline{\chi _2}\Big (\frac{g_1}{g_4}\Big ) \chi _2\Big (\frac{h}{g_4}\Big ) \nonumber \\  &= \frac{q^n\overline{\chi _2}(-1)}{|\ell _2|} \sum _{g \in \mathcal {M}_{\leqslant [(d-1)/2]}} \frac{1}{|g|} \frac{1}{2 \pi i} \oint _{|u|=q^{-\epsilon }} \sum _{g_1 \mid g} \frac{\overline{\chi _2}({g}/{g_1}) du }{|g_1|(1-u)^2u^{n+1-2\deg (g_1 \ell _2)}} \nonumber \\  &\quad \times \sum _{g_4|(g_1,h)} |g_4| \mu \Big (\frac{g_1}{g_4}\Big ) \overline{\chi _2}\Big (\frac{g_1}{g_4}\Big ) \chi _2\Big (\frac{h}{g_4}\Big ). \end{aligned}$$

When $f+h$ is not monic, $M_{2}$ in (87) does not change for $m>n$ and is multiplied by $\chi _2({{\,\textrm{sgn}\,}}(h))\overline{\chi _2}({{\,\textrm{sgn}\,}}(h)+1)$ for $m=n$.

Now, we write $g = g_1B$ and $g_1 = g_4 A$ to get

$$\begin{aligned} M_2&= \frac{q^n\overline{\chi _2}(-1)}{|\ell _2|} \sum _{g_4|h} \frac{\chi _2(h/g_4)}{|g_4|} \frac{1}{2 \pi i} \oint _{|u|=q^{-\epsilon }} \sum _{\deg (AB)<d/2-\deg (g_4)} \frac{u^{2\deg (g_4 A)} \overline{\chi _2}(AB) \mu (A) ~du}{|A^2B| u^{n+1-2\deg (\ell _2)}(1-u)^2} \\&= \frac{q^n\overline{\chi _2}(-1)}{|\ell _2|} \frac{1}{(2 \pi i)^3} \oint _{|y|=q^{-\epsilon }} \oint _{|x|=q^{-\epsilon }} \oint _{|u|=q^{-\epsilon }} \sum _{g_4|h} \frac{\chi _2(h/g_4) u^{2\deg (g_4 \ell _2)}}{|g_4|} \\&\quad \times \frac{\mathcal {L} ( {x}/{q},\overline{\chi _2}) }{\mathcal {L} ( {xyu^2}/{q^2},\overline{\chi _2}) (1-u)^2 (1-x)(1-y) (xy)^{[(d-1)/{2}]-\deg (g_4)}u^{n}} \frac{du}{u} \frac{dx}{x} \frac{dy}{y}, \end{aligned}$$

where we wrote $g=g_1B$ and $g_1=g_4B$.

In the integral over *x* we shift the contour to $|x|=q$ and encounter a pole at $x=1$. The integral over the new contour will be bounded by $q^{n-d/2-\deg (\ell _2)+\epsilon d}$.

It follows that

$$\begin{aligned} M_2&= \frac{q^n \overline{\chi _2}(-1) L(1,\overline{\chi _2})}{|\ell _2|} \sum _{g_4|h} \frac{\chi _2(h/g_4) }{|g_4|} \\  &\times \frac{1}{(2 \pi i)^2} \oint _{|y|=q^{-\epsilon }} \oint _{|x|=q^{-\epsilon }} \frac{u^{2\deg (g_4 \ell _2)}}{\mathcal {L} ( {yu^2}/{q^2},\overline{\chi _2}) (1-u)^2 (1-y) y^{[(d-1)/2]-\deg (g_4)}u^{n}} \frac{du}{u}\frac{dy}{y} \\&+ O \Big ( q^{n-d/2-\deg (\ell _2)+\epsilon d}\Big ). \end{aligned}$$

In the integral over *y* we shift the contour of integration to $|y|=q$, and we encounter the pole at $y=1$. The integral over the new contour will be bounded by $q^{n-d/2-\deg (\ell _2)+ \epsilon d}$, and we get that

$$\begin{aligned} M_2= &   \frac{q^n \overline{\chi _2}(-1) L(1,\overline{\chi _2})}{|\ell _2|} \sum _{g_4|h} \frac{\chi _2(h/g_4) }{|g_4|} \\    &   \qquad \quad \times \frac{1}{2 \pi i} \oint \frac{u^{2\deg (g_4 \ell _2)} }{\mathcal {L} ({u^2}/{q^2},\overline{\chi _2} ) (1-u)^2 u^{n}} \frac{du}{u} + O \Big ( q^{n-d/2-\deg (\ell _2)+\epsilon d} \Big ). \end{aligned}$$

We further shift the contour to $|u|=q^{1/2}$ and encounter the double pole at $u=1$. The integral over the new contour will be bounded by $q^{n/2+\epsilon d}$. Computing the residue at $u=1$ we get that

88 $$\begin{aligned}  &   M_2 = \frac{q^n \overline{\chi _2}(-1) L(1,\overline{\chi _2})}{|\ell _2|L(2,\overline{\chi _2})} \sum _{\begin{array}{c} g_4|h \\ \deg (g_4) \leqslant n/2-\deg (\ell _2) \end{array}} \frac{\chi _2(h/g_4) }{|g_4|} \nonumber \\    &   \times \Big (n+1-2\deg (g_4 \ell _2) - \frac{2\,L'(2,\overline{\chi _2})}{(\log q) L(2,\overline{\chi _2})} \Big ) + O \Big ( q^{n-d/2-\deg (\ell _2)+\epsilon d} +q^{n/2+\epsilon d} \Big ). \end{aligned}$$

## Setting up the proof when $\ell _2=1$ and the main terms

Here, we will consider the correlations

$$\begin{aligned} \sum _{f \in \mathcal {M}_n} r_{\chi _1}(f) d(f+h). \end{aligned}$$

We write

$$d(f+h) = 2 \sum _{\begin{array}{c} g \in \mathcal {M}_{\leqslant [d/2]}\\ g|(f+h) \end{array}}1 - \sum _{\begin{array}{c} g \in \mathcal {M}_{d/2}\\ g|(f+h) \end{array}} 1,$$

so

$$\begin{aligned} \sum _{f \in \mathcal {M}_n}r_{\chi _1}(f) d(f+h)= 2 \sum _{g \in \mathcal {M}_{\leqslant [d/2]}} \sum _{\begin{array}{c} f \in \mathcal {M}_n \\ f \equiv -h \hspace{0.05em}(\textrm{mod}\,g) \end{array}} r_{\chi _1}(f) - \sum _{g \in \mathcal {M}_{d/2}} \sum _{\begin{array}{c} f \in \mathcal {M}_n \\ f \equiv -h \hspace{0.05em}(\textrm{mod}\,g) \end{array}} r_{\chi _1}(f). \end{aligned}$$

We note that the second term above vanishes if *d* is odd. We then write

$$\begin{aligned} \sum _{f \in \mathcal {M}_n} r_{\chi _1}(f) d(f+h) = 2S_1 - S_2, \end{aligned}$$

and the term $S_1$ above is the same as that given in (57) when $\chi _2=1$ (after detecting the congruence condition using additive characters). We will only present the details for the evaluation of $S_1$, since $S_2$ is very similar. As before, we write $S_{11}= M_{11}+E_{12}$, and $S_{12}=M_{12}+E_{12}$, where $S_{11}$ and $S_{12}$ correspond to the sums with $\ell _1|g_1$ and $\ell _1 \not \mid g_1$ (in equation (57)). If $\ell _1 =1$, then we only have one term, and we write $S_1=M_1+E_1$. We rewrite the error terms here as (see equations (60) and (62)):89where $\mu =2\deg (g_1)+\deg (\ell _1)-n-2$, and90where $\nu =2\deg (g_1)-n-2$

If $\ell _1=1$, then the error term becomes (see Eq. (70)):91where $\mu =2\deg (g_1)-n-2$.

### Remark 7.1

As before, we remark that $E_{11}=0$ in the following cases:

- when $m \leqslant n$, *n* is even, and $\deg (\ell _1)=1$;
- when $m \leqslant n$, *n* is odd, and $\deg (\ell _1) <3$;
- when $m=n+1$, *n* is even, and $\deg (\ell _1)=1$.

Note that $E_{12}=E_1=0$ when $m < n+2$ or when $m=n+2$ and *n* is odd.

### The case $\ell _1 \ne 1$

Similarly as in equation (74), we have

$$\begin{aligned} M_{11}&= q^n L(1,\chi _1) \sum _{g_2|h} \frac{\chi _1 (g_2)}{|g_2|} \sum _{\deg (AB) \leqslant [d/2]-\deg (g_2)} \frac{ \mu (B) \chi _1 (B) }{|AB^2|} \nonumber \\&= q^n L(1,\chi _1) \frac{1}{(2 \pi i)^2} \oint _{|y|=q^{-\epsilon }} \oint _{|x|=q^{-\epsilon }} \frac{ 1}{\mathcal {L}({x}/{q^2},\chi _1)(1-xy) (1-x)(1-y)(xy)^{[d/2]}} \nonumber \\&\times \sum _{g_2|h} \frac{ \chi _1(g_2) (xy)^{\deg (g_2)}}{|g_2|} \frac{dx}{x} \frac{dy}{y}, \end{aligned}$$

Note that we can evaluate the integral over *y* exactly, and it will be given by the residues at $y=1$ and $y=1/q$. We then get that

$$\begin{aligned} M_{11}&= q^n L(1,\chi _1) \frac{1}{2 \pi i} \oint _{|x|=q^{-\epsilon }} \frac{1}{(1-x)^2 \mathcal {L}(x/q^2,\chi _1)x^{[d/2]}} \sum _{g_2|h} \frac{ \chi _1(g_2) x^{\deg (g_2)}}{|g_2|} \, \frac{dx}{x}\\&\quad - q^n L(1,\chi _1)\sum _{g_2|h} \frac{ \chi _1(g_2) }{|g_2|} \frac{1}{2 \pi i} \oint _{|y|=q^{-\epsilon }} \frac{1}{ \mathcal {L} ( x/q^2,\chi _1)(1-x)^2} dx . \end{aligned}$$

Note that the second integral above vanishes, because the integrand is analytic inside the circle of integration $|x|=q^{-\epsilon }$. In the first integral, we shift the contour of integration to $|x|=q$ and obtain an error term of size $q^{n-d/2+\epsilon d}$. The main term will be given by the residue at $x=1$, so

92 $$\begin{aligned} M_{11}&= \frac{q^n L(1,\chi _1)}{L(2,\chi _1)} \sum _{\begin{array}{c} g_2|h \\ \deg (g_2) \leqslant [d/2] \end{array}} \frac{\chi _1(g_2)}{|g_2|} \left( [d/2]+1 - \deg (g_2) - \frac{L'(2,\chi _1)}{(\log q) L(2,\chi _1)}\right) \nonumber \\&\quad + O \Big ( q^{n-d/2+\epsilon d}\Big ). \end{aligned}$$

The evaluation of $M_{12}(\chi _1,1)$ is similar to that of $M_{12}(\chi _1,\chi _2)$ in the previous sections. We skip some of the details, and we get that

$$\begin{aligned} M_{12}&= \frac{q^n L(1,\overline{\chi _1})}{|\ell _1|} \sum _{g_4|h} \frac{\chi _1(h/g_4)}{|g_4|}\frac{1}{2 \pi i} \oint _{|u|=q^{-\epsilon }} \frac{1}{\mathcal {L}(u/q^2,\overline{\chi _1} )(1-u)^2u^{[d/2]-\deg (\ell _1) - \deg (g_4)}} \frac{du}{u}. \end{aligned}$$

We shift the integral to $|u|=q$ and encounter a pole at $u=1$. The integral over the new contour will be bounded by $q^{n-d/2+\epsilon d}$. We have

93 $$\begin{aligned} M_{12}&= \frac{q^n L(1,\overline{\chi }_1)}{|\ell _1|L(2,\overline{\chi }_1)} \sum _{\begin{array}{c} g_4 | h \\ \deg (g_4) \leqslant [d/2]-\deg (\ell _1) \end{array}} \frac{ \chi _1(h/g_4)}{|g_4|} \Big [[d/2]+1-\deg (g_4\ell _1) - \frac{L'(2,\overline{\chi }_1)}{(\log q) L(2,\overline{\chi }_1)} \Big ]\nonumber \\&\quad + O \Big ( q^{n-d/2+\epsilon d}\Big ). \end{aligned}$$

For the other term

$$\begin{aligned} \sum _{g \in \mathcal {M}_{d/2}} \sum _{\begin{array}{c} f \in \mathcal {M}_n \\ f \equiv -h \hspace{0.05em}(\textrm{mod}\,g) \end{array}} r_{\chi _1}(f), \end{aligned}$$

which exists when $2\mid d$, we have

$$\begin{aligned} M_{21}&= 1\!\!1_{2|d} q^n L(1,\chi _1) \sum _{g_2|h} \frac{\chi _1 (g_2)}{|g_2|} \sum _{\deg (B) \leqslant d/2-\deg (g_2)} \frac{\mu (B) \chi _1 (B) }{|B^2|} \sum _{\deg (A)=d/2-\deg (g_2)-\deg (B)}\frac{1}{|A|}. \end{aligned}$$

We get that

94 $$\begin{aligned} M_{21} = 1\!\!1_{2|d} \frac{q^n L(1,\chi _1)}{L(2,\chi _1)} \sum _{\begin{array}{c} g_2|h \\ \deg (g_2) \leqslant d/2 \end{array}} \frac{\chi _1(g_2)}{|g_2|} + O \Big ( q^{n-d/2+\epsilon d} \Big ). \end{aligned}$$

Finally, we have

95 $$\begin{aligned} M_{22}&= 1\!\!1_{2|d} \frac{q^n L(1,\overline{\chi _1})}{|\ell _1|} \sum _{g_4|h} \frac{ \chi _1(h/g_4)}{|g_4|}\frac{1}{2 \pi i} \oint _{|u|=q^{-\epsilon }} \frac{du}{\mathcal {L}(u/q^2,\overline{\chi _1} )(1-u)u^{1+d/2-\deg (\ell _1) - \deg (g_4)}} \nonumber \\&= \frac{q^n L(1,\overline{\chi _1})}{|\ell _1|L(2,\overline{\chi _1})} \sum _{\begin{array}{c} g_4|h \\ \deg (g_4) \leqslant d/2-\deg (\ell _1) \end{array}} \frac{\chi _1(h/g_4)}{|g_4|} + O \Big (q^{n-d/2+\epsilon d}\Big ). \end{aligned}$$

### The case $\ell _1=1$

Finally, we consider the correlations of *d*(*f*) and $d(f+h)$. Similarly as before (see equation (85)), we have

$$\begin{aligned} M_{1} = \frac{q^n}{(2 \pi i)^3} \oint _{|y|=q^{-\epsilon }} \oint _{|x|=q^{-\epsilon }} \oint _{|u|=q^{-\epsilon }} \frac{1-xyu^2/{q}}{ (1-u)^2 (1-x)^2(1-y)(xy)^{[d/2]}u^{n}}\\ \times \sum _{g_2|h} \frac{(xyu^2)^{\deg (g_2)}}{|g_2| } \frac{du}{u} \frac{dx}{x} \frac{dy}{y}. \end{aligned}$$

We note that the integral over *y* above is precisely given by the residue of the pole at $y=1$. We then get that

$$\begin{aligned} M_{1}&= \frac{q^n}{(2 \pi i)^2} \oint _{|x|=q^{-\epsilon }} \oint _{|u|=q^{-\epsilon }} \frac{1-xu^2/{q}}{ (1-u)^2 (1-x)^2x^{[d/2]}u^{n}} \sum _{g_2|h} \frac{(xu^2)^{\deg (g_2)}}{|g_2| } \frac{du}{u} \frac{dx}{x}. \end{aligned}$$

Note that in order for the integral over *u* not to vanish we need $\deg (g_2) \leqslant [n/2]$. We similarly have that

$$\begin{aligned} M_2&= 1\!\!1_{2|d} \frac{q^n}{(2 \pi i)^2}\oint _{|x|=q^{-\epsilon }} \oint _{|u|=q^{-\epsilon }} \frac{ 1-{xu^2}/{q}}{(1-u)^2 (1-x)x^{[d/2]}u^{n}} \sum _{g_2|h} \frac{(xu^2)^{\deg (g_2)}}{|g_2| } \frac{du}{u} \frac{dx}{x}. \end{aligned}$$

The integrals above are given by the residue of the poles at $u=1$ and $x=1$ (with no error terms), and computing both of the terms and putting them together, we get that the final main term *M* is given by

96 $$\begin{aligned} M= &   2q^n\sum _{\begin{array}{c} g_2|h \\ \deg (g_2) \leqslant [n/2] \end{array}} \frac{1}{|g_2|} \Bigg [2\deg (g_2)^2\Big (1-\frac{1}{q}\Big )\nonumber \\    &   \quad \quad - \deg (g_2) \bigg (\Big (3+\frac{1}{q}\Big )+\Big (1-\frac{1}{q}\Big )(n+2[d/2]) \bigg )\nonumber \\  &   \quad \quad + \bigg ( n+1+[d/2] \Big ( n\Big (1-\frac{1}{q}\Big ) + \Big (1+\frac{1}{q}\Big ) \Big ) \bigg ) \Bigg ] \nonumber \\  &   \quad - 1\!\!1_{2|d}q^n \Bigg (\sum _{\begin{array}{c} g_2|h \\ \deg (g_2) \leqslant [(n-2)/2] \end{array}} \frac{1}{|g_2|} \Bigg [ (n-2\deg (g_2)) \Big ( 1-\frac{1}{q}\Big )+1+\frac{1}{q}\Bigg ]\nonumber \\  &   \quad \quad \quad +\sum _{\begin{array}{c} g_2|h \\ \deg (g_2)= [n/2] \end{array}} \frac{1}{|g_2|}(n-2\deg (g_2)+1) \Bigg ). \end{aligned}$$

## Bounding the error terms

Combining Remarks 5.1, 5.2, 5.4, 5.5, 5.7, 5.8, 5.6, and 7.1, we see that when $\deg (h) \leqslant n$ and $\deg (\ell _1)<2$ and $2 \deg (\ell _2)+\deg (\ell _1)<4$, the various error terms given by (60), (62), (64), (66), (68), (70), (72), (89), (90), (91) vanish and the only error terms in the asymptotic formulas are those in the main term computations (if any). In particular, if $(\deg (\ell _1), \deg (\ell _2)) \in \{ (0,0), (0,1), (1,0),(1,1)\}$ and $\deg (h) \leqslant n$, then the error terms mentioned above vanish.

In fact, we can say a little more. Namely, we obtain exact formulas in the following scenarios as well. Let $m=n+a$ for $a\geqslant 0$, where we recall that $m=\deg (h)$. Then the error terms $E_{ij}$ and $E_i$ for $i=1,2$ and $j=1,2$ vanish in the following cases:

$$ (a,\deg (\ell _1),\deg (\ell _2)) \in {\left\{ \begin{array}{ll} (0,0,0)&  \\ (0,0,1) &  \\ (0,1,0) &  \\ (0,2,0) &  \text{ and } n\equiv 1 \hspace{0.05em}(\textrm{mod}\,2) \\ (0,1,1) &  \text{ and } n \equiv 0 \hspace{0.05em}(\textrm{mod}\,2) \\ (1,0,0) &  \\ (1,1,0) &  \text{ and } n \equiv 0 \hspace{0.05em}(\textrm{mod}\,2) \\ (1,0,1) &  \text{ and } n \equiv 1 \hspace{0.05em}(\textrm{mod}\,2) \\ (2,0,0) &  \text{ and } n \equiv 1 \hspace{0.05em}(\textrm{mod}\,2). \end{array}\right. }$$

Now we focus on bounding the various error terms when we are not in one of the scenarios described above. We will only bound a few of them, since they are all similar. We first bound the terms $E_{11}$ and $E_1$, given in (60) and (91) (they are very similar, the only difference being that in the latter case, $\chi _1=\chi _2=1$.) We interchange the sums over $g_1$ and *g* writing $g=g_1g_2$ and we have

$$\begin{aligned} E_{11}&= \frac{q^nG(\chi _1)}{|\ell _1|} \sum _{g_1\in \mathcal {M}_{\leqslant [d/2]}} \frac{ \chi _1 \chi _2(g_1)}{|g_1|^2} \sum _{g_2 \in \mathcal {M}_{\leqslant [d/2]-\deg (g_1)}} \frac{\chi _2(g_2)}{|g_2|}\\  &\times \sum _{\lambda \in \mathbb {F}_q^\times } \sum _{\eta _1,\eta _2 \in \{0,1\}} \sum _{0\leqslant k \leqslant \mu } b_{\mu -k}(\overline{\chi }_1) \sum _{f \in \mathcal {M}_k} r_{\overline{\chi }_1}(f) S(h,\lambda f; g_1), \end{aligned}$$

where $\mu = 2\deg (g_1) + \deg (\ell _1)-n-2$ and we recall that *S*(*a*, *b*; *c*) is the function field Kloosterman sum given by (15). We further write

$$\begin{aligned} E_{11}&= \frac{q^nG(\chi _1)}{|\ell _1|} \sum _{j=0}^{[d/2]} q^{-2j} \sum _{g_2 \in \mathcal {M}_{\leqslant [d/2]-j}} \frac{\chi _2(g_2)}{|g_2|} \sum _{\lambda \in \mathbb {F}_q^\times }\sum _{\eta _1,\eta _2 \in \{0,1\}} \\&\times \sum _{k=0}^{ 2j + \deg (\ell _1)-n-2} b_{2\deg (g_1)+\deg (\ell _1)-n-2-k}(\overline{\chi }_1) \sum _{f \in \mathcal {M}_k} r_{\overline{\chi }_1}(f) \sum _{g_1 \in \mathcal {M}_j} \chi _1 \chi _2(g_1) S(h,\lambda f; g_1). \end{aligned}$$

In the case when $\chi _1 \chi _2 = 1$ (namely, when $\ell _1=\ell _2=1$) we use the Linnik–Selberg theorem (18) and we have that

$$\begin{aligned} \sum _{g_1 \in \mathcal {M}_j} S(h,\lambda f; g_1) \ll q^{j+\epsilon j} |fh|^{\epsilon }. \end{aligned}$$

Trivially bounding the other sums, we get that in this case,

97 $$\begin{aligned} E_{11} \ll q^{d/2+\epsilon d}. \end{aligned}$$

If we are not in the case mentioned above, then we use the Weil bound (17) on the sum over $g_1$ and we have that

$$\begin{aligned} |S(h,\lambda f;g_1)| \leqslant q^{\deg (g_1)/2+\deg ( (f,h,g_1))/2} 2^{\omega (g_1)}. \end{aligned}$$

Let $$A=(f,h)$$. Then

$$\begin{aligned} \sum _{g_1 \in \mathcal {M}_j} 2^{\omega (g_1)} q^{\deg (g_1)/2+\deg ((A,g_1))/2} \ll q^{j/2+\epsilon j} \sum _{R|A} \sum _{\begin{array}{c} g_1 \in \mathcal {M}_j \\ (g_1,A)=R \end{array}} 1 \ll q^{3j/2+\epsilon j} |A|^{\epsilon } \ll q^{3j/2+\epsilon j+\epsilon d}. \end{aligned}$$

Trivially bounding all the other sums, we get that

98 $$\begin{aligned} E_{11} \ll q^{3d/4+\deg (\ell _1)/2+\epsilon d}. \end{aligned}$$

Now we focus on bounding $E_{21}$, given in (64) (in the case $\ell _1 \ne \ell _2)$.) We rewrite it aswhere $\mu = 2 \deg (g_1 \ell _2)+\deg (\ell _1)-n-2$. Now we rewrite

$$\begin{aligned} G(\chi _2) \overline{\chi _2}(-b_1) = \sum _{a \hspace{0.05em}(\textrm{mod}\,\ell _2)} \chi _2(a) e \Big ( - \frac{ab_1}{\ell _2}\Big ), \end{aligned}$$

so we have that

$$\begin{aligned} E_{21}&= \frac{q^n \chi _1(\ell _2)G(\chi _1) }{|\ell _1 \ell _2^2|} \sum _{j=0}^{[(d-1)/2]} q^{-2j}\sum _{g_2 \in \mathcal {M}_{\leqslant [(d-1)/2]-j}} \frac{\overline{\chi _2}(g_2)}{|g_2|} \sum _{\lambda \in \mathbb {F}_q^\times } \sum _{\eta _1,\eta _2 \in \{0,1\}}\\&\times \sum _{0\leqslant k \leqslant \mu } b_{\mu -k}(\overline{\chi _1}) \sum _{f \in \mathcal {M}_k} r_{\overline{\chi }_1}(f) \sum _{a \hspace{0.05em}(\textrm{mod}\,\ell _2)}\chi _2(a)\sum _{g_1 \in \mathcal {M}_j} \chi _1(g_1) S(h-ag_1,\lambda f; g_1 \ell _2). \end{aligned}$$

Now we use the Weil bound (17) on the Kloosterman sum and we have that

$$\begin{aligned} |S(h-ag_1,\lambda f;g_1 \ell _2)|&\leqslant q^{\deg (g_1 \ell _2)/2+\deg ((h-ag_1,f,g_1\ell _2))/2} 2^{\omega (g_1 \ell _2)} \\&\ll q^{\deg (g_1 \ell _2)/2+\deg ((f,g_1\ell _2))/2} |g_1 \ell _2|^{\epsilon }. \end{aligned}$$

It follows that introducing the sum over *f* and trivially bounding everything else,

$$\begin{aligned}  &   \sum _{f \in \mathcal {M}_k} r_{\overline{\chi }_1}(f)\sum _{g_1 \in \mathcal {M}_j} \chi _1(g_1) S(h-ag_1,\lambda f; g_1 \ell _2) \\    &   \qquad \qquad \quad \ll |\ell _2|^{\epsilon } q^{\deg (\ell _2)/2+j/2+\epsilon j} \sum _{\begin{array}{c} f \in \mathcal {M}_k \\ \ell _2|f \end{array}} \sqrt{|\ell _2|} \sum _{g_1 \in \mathcal {M}_j} q^{\deg ((f/\ell _2,g_1))/2} \\    &   \quad \quad +|\ell _2|^{\epsilon } q^{\deg (\ell _2)/2+j/2+\epsilon j} \sum _{\begin{array}{c} f \in \mathcal {M}_k \\ \ell _2 \not \mid f \end{array}} \sum _{g_1 \in \mathcal {M}_j} q^{\deg ((f,g_1))/2} \ll q^{3j/2+\epsilon j+\deg (\ell _2)/2} |\ell _2 |^{\epsilon } q^{k(1+\epsilon )}. \end{aligned}$$

We trivially bound all the remaining sums, and we get that

99 $$\begin{aligned} E_{21} \ll q^{3d/4+\epsilon d+\deg (\ell _1)/2+3\deg (\ell _2)/2}. \end{aligned}$$

## Proof of the main theorems

Finally, we are ready to prove Theorems 1.5, 1.1, 1.8, 1.9, 1.10.

Using Remark 5.3 and equations (75), (84), (98) finishes the proof of Theorem 1.5.

Now using equations (96), (97) finishes the proof of Theorem 1.1.

Using equations (75), (77), (80), (82), and (99) finishes the proof of Theorem 1.8.

Using (86),(88), and (99) finishes the proof of Theorem 1.9.

Finally, combining (92), (93), (94), (95), (99) finishes the proof of Theorem 1.10.

To deduce Corollary 1.4, we simply set $\chi _1 = \chi _2 = \chi$ in Theorem 1.5 and note that the quadratic character modulo *T* is an odd character. In that case, we have $\chi (-1)G(\chi )^2G(\chi ^2) = -q$.

## Data Availability

This article has no associated data.
